# The multi-organ landscape of B cells highlights dysregulated memory B cell responses in Crohn's disease

**DOI:** 10.1093/nsr/nwaf009

**Published:** 2025-01-10

**Authors:** Dianyu Chen, Song Xu, Shuyan Li, Qiuying Wang, Hui Li, Danyang He, Yan Chen, Heping Xu

**Affiliations:** Westlake Laboratory of Life Sciences and Biomedicine, Hangzhou 310024, China; Laboratory of Systems Immunology, School of Medicine, Westlake University, Hangzhou 310024, China; Key Laboratory of Growth Regulation and Translational Research of Zhejiang Province, School of Life Sciences, Westlake University, Hangzhou 310024, China; Department of Gastroenterology, The Second Affiliated Hospital, Zhejiang University School of Medicine, Hangzhou 310000, China; Department of Nursing, The Second Affiliated Hospital, Zhejiang University School of Medicine, Hangzhou 310009, China; Westlake Laboratory of Life Sciences and Biomedicine, Hangzhou 310024, China; Laboratory of Systems Immunology, School of Medicine, Westlake University, Hangzhou 310024, China; Key Laboratory of Growth Regulation and Translational Research of Zhejiang Province, School of Life Sciences, Westlake University, Hangzhou 310024, China; Westlake Laboratory of Life Sciences and Biomedicine, Hangzhou 310024, China; Laboratory of Systems Immunology, School of Medicine, Westlake University, Hangzhou 310024, China; Key Laboratory of Growth Regulation and Translational Research of Zhejiang Province, School of Life Sciences, Westlake University, Hangzhou 310024, China; Laboratory of Systems Immunology, School of Medicine, Westlake University, Hangzhou 310024, China; Key Laboratory of Growth Regulation and Translational Research of Zhejiang Province, School of Life Sciences, Westlake University, Hangzhou 310024, China; Center for Inflammatory Bowel Diseases, Department of Gastroenterology, The Second Affiliated Hospital, Zhejiang University School of Medicine, Hangzhou 310009, China; Westlake Laboratory of Life Sciences and Biomedicine, Hangzhou 310024, China; Laboratory of Systems Immunology, School of Medicine, Westlake University, Hangzhou 310024, China; Key Laboratory of Growth Regulation and Translational Research of Zhejiang Province, School of Life Sciences, Westlake University, Hangzhou 310024, China

**Keywords:** Crohn's disease, inflammation, scRNA-seq, scBCR-seq, B cell differentiation, plasma cells, memory B cells

## Abstract

Crohn's disease (CD) is a prevalent type of inflammatory bowel disease (IBD) with dysregulated antibody responses. However, there is a lack of comprehensive analysis of B cell responses in CD. Here, we collected B cells from the small intestine, colon and blood of CD patients and control subjects. Through the coupled analysis of transcriptome and immunoglobulin (Ig) gene in individual cells, we characterized the cellular composition, transcriptome and Ig clonotype in different B cell subtypes. We observed shared disruptions in plasma cell (PC) responses between different IBD subtypes. We revealed heterogeneity in memory B cells (MBCs) and showed a positive correlation between gut resident-like MBCs and disease severity. Furthermore, our clonotype analysis demonstrated an increased direct differentiation of MBCs into PCs in CD patients. Overall, this study demonstrates significantly altered B cell responses associated with chronic inflammation during CD and highlights the potential role of mucosal MBCs in CD pathogenesis.

## INTRODUCTION

Inflammatory bowel disease (IBD) includes Crohn's disease (CD) and ulcerative colitis (UC), both of which are immune-mediated chronic disorders affecting the gastrointestinal (GI) tract [1]. While UC primarily involves inflammation and tissue damage in the colonic epithelium, CD is characterized by transmural segmental inflammation, particularly in the colon and terminal ileum (TI) [2,3]. Recent advances in single-cell RNA sequencing (scRNA-seq) profiling have shed light on the cellular landscape of the colon in CD and UC, revealing both shared and distinct abnormalities between the two diseases [4–7]. The specific profiling of the immune cell compartment further highlighted dysregulated plasma cell (PC) responses in the colon of UC patients [8–10]. PC infiltration and expansion is a well-known pathological hallmark of UC and is linked with the risk of disease recurrence [2,11]. Additionally, a low-affinity IgG Fc receptor variant has been found to be protective against UC [12], suggesting an important function of IgG antibodies in UC pathogenesis. Notably, CD is also associated with the dysregulated anti-microbial IgG and IgA responses [13,14], yet comprehensive single-cell transcriptional profiling of B cell responses in CD is lacking. Moreover, given that some of the CD-associated alterations are restricted to either the colon or the small intestine [4], a detailed analysis of B cell responses in both the small intestine and colon is needed to understand the tissue-specific humoral immune responses associated with IBD.

Intestinal B-lineage cells consist of naïve B cells (NBCs) and three antigen-experienced B cell subtypes, including germinal center (GC) B cells (GCBCs), memory B cells (MBCs), and PCs. In addition to PCs, aberrant GCBC and NBC responses are also observed in UC patients, including the expansion of NBCs in inflamed colon regions and impaired GCBC responses indicated by reduced Ig somatic hypermutation (SHM) [9,13,14]. MBCs play an essential role in driving the adaptation of secretory antibodies to gut microbiota [15]. However, their heterogeneity and responses in the intestinal mucosa in both UC and CD remain poorly understood. Recent findings in human intestines have indicated the presence of resident-like MBCs highly expressing CD69 [16,17], a marker associated with tissue-resident MBCs in mouse lungs after respiratory infection [18]. It's known that MBCs can either differentiate into PCs or re-enter the GC for additional rounds of proliferation and selection [19]. Lung-resident MBCs have been suggested to differentiate into PCs *in situ* for rapidly increasing local antibody concentrations in response to the secondary infection [16–18]. However, it is unknown whether the fate of MBCs in the intestine is dysregulated by the chronic inflammation in CD patients.

In this study, we conducted scRNA-seq and V(D)J-seq analysis of matched biopsies of TI and colon, as well as blood samples from CD patients and non-IBD subjects, to comprehensively analyze B cell transcriptional states and the B cell receptor (BCR) repertoire. By integrating transcriptomic data with antigen-binding-induced BCR modifications, including SHM and class-switch recombination (CSR), we identified major subtypes of antigen-experienced B cells in both the intestine and circulation. Our analysis revealed profound yet distinct changes in the transcriptional states of GCBCs, PCs and MBCs in CD. The proportions of circulating PCs and resident-like MBCs in the TI were positively correlated with the severity of mucosal inflammation in CD patients. We observed a significant reduction of IgA^+^ cells in both PCs and MBCs in CD, whereas the CSR of GCBCs remained unchanged. The expansion of IgG^+^ PCs in CD was observed, akin to the findings in UC [8,9]. MBCs exhibited increased IgM^+^ cells in CD patients compared to non-IBD subjects, highlighting differential perturbations in CSR between MBCs and PCs in CD. Furthermore, analysis of BCR mutations revealed an impaired SHM in all antigen-experienced B cell subsets, reflecting an impaired GC response in CD. Clonotype analysis of each B cell subset revealed increased clonal expansion in MBCs, especially resident-like MBCs in the TI. Finally, a comparison of clonotype similarity across different B cell subsets demonstrated increased similarity between MBCs and PCs in CD, suggesting an increased direct differentiation of MBCs into PCs. Altogether, our analysis of cellular and transcriptomic changes associated with CD in antigen-experienced B cells provides valuable insights into the highly dysregulated B cell response in CD and highlights the potential role of MBCs in CD pathogenesis.

## RESULTS

### Heterogeneity of antigen-experienced human B cells

**Figure 1.**
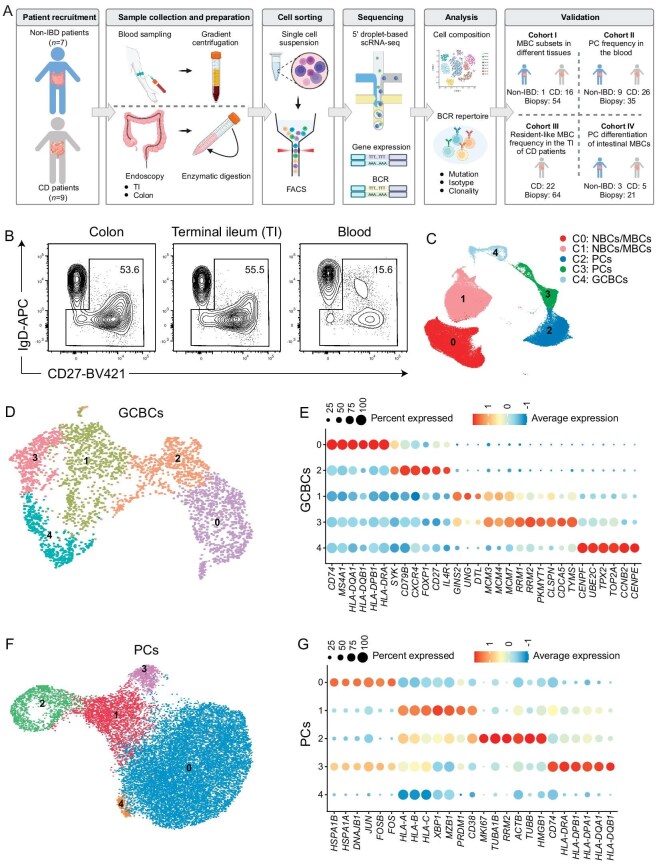
scRNA-seq atlas of antigen-experienced B cells in CD patients and control subjects. (A) A schematic diagram of the experimental design. (B) Representative flow plots showing the gating strategies for IgD^–^ or CD27^+^ cells in CD19^+^ cells (Fig. S1B) in the colon, terminal ileum (TI), and blood. (C) Uniform manifold approximation and projection (UMAP) visualization of transcriptome profiles of major B cell clusters. Cells are colored and numbered by cluster membership. NBCs: naïve B cells, MBCs: memory B cells, PCs: plasma cells, GCBCs: germinal center B cells. (D) UMAP visualization of 5 clusters of GCBCs. Cells are colored and numbered by cluster membership. (E) Representative marker genes (columns) across GCBC clusters (rows) in (D). The fraction of cells in the clusters expressed a gene (dot size) and the Z score of mean expression (log_2_(TP10K + 1)) of this gene in the cluster. (F) UMAP visualization of 5 clusters of PCs. Cells are colored and numbered by cluster membership. (G) Representative marker genes (columns) across PC clusters (rows) in (F). The fraction of cells in the clusters expressed a gene (dot size) and the Z score of mean expression (log_2_(TP10K + 1)) of this gene in the cluster.

We collected a total of 61 samples from 16 patients at the Second Affiliated Hospital Zhejiang University School of Medicine (Fig. 1A, Fig. S1A and Table S1). Among these patients, there were 8 individuals with active CD who exhibited segmental inflammation in the terminal ileum (TI). For these active CD patients, we collected biopsies from both visibly inflamed and non-inflamed regions in the TI (Fig. S1A). We also collected biopsies from the non-inflamed regions of the TI from 1 CD patient in endoscopic remission and 7 non-IBD patients. In addition to the TI biopsies, we collected matched non-inflamed colon biopsies and blood samples from all subjects (Fig. S1A). After the preparation of single-cell suspensions, we performed fluorescence-activated cell sorting (FACS) to enrich antigen-experienced B cells (CD45^+^CD19^+^IgD^–^ or CD27^+^) from each sample (Fig. 1B and Fig. S1B). Additionally, naïve B cells (CD45^+^CD19^+^IgD^+^CD27^–^) from the blood samples of 5 patients were also purified by FACS (Fig. S1C). All flow-sorted B cells were profiled through 5′ directed single-cell RNA sequencing (scRNA-seq) for both mRNA and paired V(D)J profiling, recovering matched single-cell V(D)J and gene expression profiles. After performing quality control of the sequencing data (see ‘Methods’), we obtained single-cell transcriptomes of 89 467 cells.

Computational analysis of the scRNA-seq initially classified cells into nine distinct cell clusters including two clusters of naïve/memory B cells (*CD19, MS4A1, BANK1* and *SELL*), three clusters of plasma cells (PBs/PCs) (*TNFRSF17, MZB1, PRDM1, IRF4* and *XBP1*), one each cluster of germinal center (GC) B cells (*AICDA, S1PR2, BCL6* and *MKI67*), macrophages (*CST3, LYZ, C1QA* and *C1QB*), T cells (*CD3E, NKG7* and *CST7*) and stromal cells (*SPARC, COL1A2* and *MYL9*) (Fig. S1D and E). Cluster 3 of PCs appeared to consist of low-quality cells, exhibiting low RNA and gene counts, as well as a high percentage of mitochondrial genes (Fig. S1F). Consequently, this cluster, along with other non-B-cell clusters, was excluded from downstream analyses (Fig. 1C). While the overall proportions of these cell clusters in each tissue were similar across all individuals (Fig. S1G), naïve/memory B cells and PCs from the intestine and blood tended to be clustered separately (Fig. S1H and I). We then separated naïve/memory B cells, PCs and GC B cells (GCBCs) *in silico*, and performed detailed clustering for each of them.

Within GCBCs isolated from the intestine biopsies (Fig. S2A and B), further analysis revealed 5 cell clusters (Fig. 1D). Cluster 0 cells exhibited the highest expression of genes involved in the major histocompatibility complex II (MHC-II) antigen presentation pathway, including *CD74* and *HLA-DQA1* (Fig. 1E). Concurrently, these cells maintained the expression of genes involved in BCR signaling, such as *SYK* and *CD79B*, which were predominantly enriched in cluster 2 cells. Conversely, cells in clusters 1, 3 and 4 showed decreased expression of these genes and instead showed elevated expression of genes related to DNA replication, chromatin segregation and cell division, including *GINS2, MCM3, TOP2A* and *CENPF*. Based on the expression patterns of these genes, clusters 1, 3 and 4 appeared to correspond to GCBCs at different phases of the cell cycle: G1→S, S→G2 and G2→M, respectively. To further analyze the mitotic state and spatial location of individual GCBCs, we calculated their cell cycle [20] and spatial localization [21] signatures. Cluster 1 and 3 cells exhibited the highest S-phase scores, while cluster 4 displayed the highest G2/M-phase score, aligning with marker gene expression (Fig. S2C). In contrast, cluster 0 and 2 cells showed significantly lower scores on both measures. Moreover, cluster 0 has the highest light zone (LZ) score but the lowest dark zone (DZ) score, while cluster 4 showed the opposite pattern (Fig. S2D). Clusters 1, 2 and 3 fell in the middle of the spectrum, likely representing cells in a transitional state between the DZ and LZ.

PCs were further partitioned into 5 clusters (Fig. 1F). Clusters 0 and 3 contained a higher proportion of cells isolated from the colons and TIs, while cluster 2 cells primarily consisted of blood-derived cells (Fig. S2E and F). Of note, cluster 3 cells exhibited the highest expression of MHC-II–related genes, such as *CD74* and *HLA-DRA1* (Fig. 1G). Cluster 2 exhibited enrichment for cells in mitosis, as indicated by the highest cell cycle signature score (Fig. S2G). Cluster 4 had a limited number of marker genes and significantly lower RNA content than the other clusters (Fig. S2H), and was therefore considered to be made up of low-quality cells and excluded from downstream analysis.

**Figure 2.**
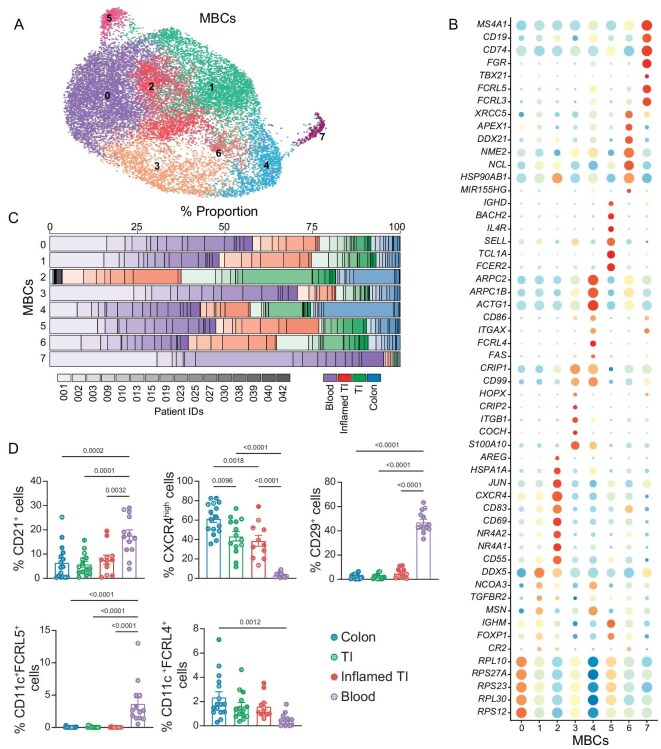
MBC heterogeneity in the intestine and circulation. (A) UMAP visualization of 8 clusters of MBCs. Cells are colored and numbered by cluster membership. (B) Representative marker genes (rows) across MBC clusters (columns) in (A). The fraction of cells in the clusters expressed a gene (dot size) and the Z score of mean expression (log_2_(TP10K + 1)) of this gene in the cluster. (C) The proportion of cells in each MBC cluster (rows) that are derived from each colon (blue), TI (green), inflamed TI (red) or blood (purple) sample. (D) Quantification of indicated MBC subpopulation percentages in total MBCs collected from the colon (*n* = 16 donors), TI (*n* = 14 donors), inflamed TI (*n* = 11 donors) or blood (*n* = 13 donors). Bars display the mean ± s.e.m. *P* values were calculated using one-way ANOVA with Tukey's multiple-comparison test.

In contrast to GCBCs and PCs, which express a distinct set of marker genes, identifying memory B cells (MBCs) can be challenging due to the lack of specific marker genes. While CD27 is commonly used to identify human MBCs, there are instances where MBCs do not express CD27 [22]. However, MBCs, along with other antigen-experienced B cells, undergo somatic hypermutation (SHM) and class switch recombination (CSR), leading to alterations in their B cell receptor (BCR) sequences. We hypothesized that these antigen-driven alterations in the BCR could aid in distinguishing antigen-experienced B cells from naïve B cells, which possess germline Ig sequences. To this end, we aligned the Ig variable region (IgV) genes in our dataset with the germline IgV genes in the IMGT database [23] (see ‘Methods’). Of note, we aligned the IgV genes of flow-sorted naïve B cells (IgD^+^CD27^–^) separately to establish a threshold for calling antigen-driven mutations (Fig. S3A–C). Subsequently, we identified MBCs *in silico* based on the presence of mutated IgV genes and/or class-switched Ig isotypes within the naïve/memory B cell clusters (Fig. S3D–F). These inferred MBCs were further analyzed and clustered into eight distinct cell subsets (Fig. 2A and B).

We found that the MBC clusters exhibited differential expression of marker genes associated with several important B cell sub-populations reported in previous studies. For instance, cluster 2 cells highly expressed activation-related genes, including *CD83* and *CD69* (Fig. 2B), with the latter recently proposed as a marker for gut-resident MBCs [17]. Intriguingly, cluster 2 also expressed *AREG*, which has been shown to be expressed by autoreactive B cells in rheumatoid arthritis patients [24]. Cluster 1 cells, characterized by an IgM^+^IgD^–^ phenotype, also expressed *CD69* and *CD83*, albeit at a lower abundance compared to cluster 2 cells. Moreover, cluster 4 and cluster 7 cells expressed *ITGAX*, a marker associated with atypical MBCs or age-associated B cells [25–27]. However, cluster 4 cells specifically expressed *FCRL4*, while cluster 7 cells exhibited higher expression levels of *FCRL3* and *FCRL5* (Fig. 2B). Cluster 3 cells showed high expression of *FAS, ITGB1, CRIP1* and *CRIP2*, representing a subset of MBCs reported in a recent study [28]. Additionally, cluster 6 cells displayed a high expression of genes involved in CSR [29], whereas cluster 5 cells showed a naïve B cell-like phenotype, expressing *IgD* and *IgM*.

We observed differential enrichment of MBCs from the intestine and blood in different cell clusters (Fig. 2C and Fig. S3G). For example, clusters 3 and 7 mainly consisted of blood cells, while cluster 2 was predominantly composed of intestinal cells. To validate these findings, we selected cell surface markers for the major MBC clusters based on the scRNA-seq data (Fig. S3H and I) and analyzed their frequencies in an independent patient cohort (Table S1, validation cohort I) using multi-parametric flow cytometry (Fig. S3J). Importantly, the cell distribution patterns of each MBC subset across different tissues, as determined by the flow cytometry, were largely consistent with the observations in the scRNA-seq dataset (Fig. 2D and Fig. S3I). Collectively, we generated a comprehensive single-cell transcriptome and Ig dataset covering diverse antigen-experienced B cell subsets in the intestine and circulation of humans in CD or non-IBD conditions.

### Cell type-specific remodeling of transcriptional states by CD

**Figure 3.**
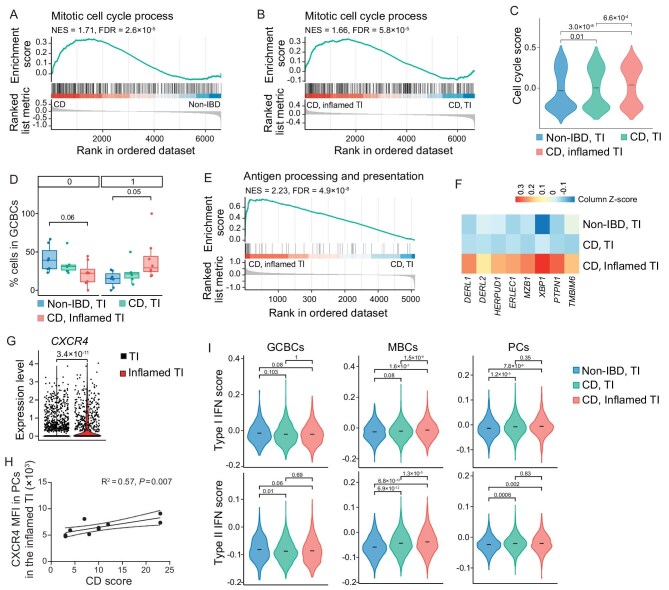
Cell-type-specific differential expression in CD. (A and B) Gene set enrichment analysis (GSEA) plots showing the enrichment of mitotic cell cycle genes in GCBCs from CD compared to non-IBD patients (A), or from the inflamed regions compared to the visibly healthy regions in the TI of CD patients (B). (C) Violin plot showing the distribution of cell cycle signature score in GCBCs from the TI of non-IBD patients (blue) and the visibly healthy (green) or inflamed (red) TI regions of CD patients. Crossbars display the means. (D) Quantification of the percentages of clusters 0 and 1 cells in total GCBCs in the TI of non-IBD patients (blue, *n* = 7) and the visibly healthy (green, *n* = 6) or inflamed (red, *n* = 8) TI regions in CD patients. (E) GSEA plot showing the enrichment of genes in the antigen processing and presentation pathway in PCs from the inflamed regions compared to the visibly healthy regions in the TI of CD patients. (F) Heatmap showing the relative expression (column-wise Z score of log_2_(TP10K + 1)) of indicated genes in PCs from the TI of non-IBD patients and the visibly healthy or inflamed TI regions of CD patients. (G) Quantification of CXCR4 expression in PCs from the visibly healthy (black) or inflamed (red) TI regions of CD patients. (H) Linear regression with 95% confidence bands showing the correlation between disease scores and median fluorescence intensity (MFI) of CXCR4 in PCs from the inflamed TI regions of CD patients (*n* = 11). (I) Violin plot showing the distribution of Type I interferon (IFN) (top) or Type II IFN (bottom) signature scores of indicated cell types from the TI of non-IBD patients (blue) and the visibly healthy (green) or inflamed (red) TI regions of CD patients. Crossbars display the means. *P* values were calculated using one-way ANOVA with Tukey's multiple-comparison test (C, D, I), Model-based Analysis of Single-cell Transcriptomics (MAST) test (G) or Pearson test (H).

We next sought to determine the impact of CD on the transcriptional states of individual B cell subsets (Table S2). In comparison to GCBCs isolated from non-IBD patients, GCBCs from CD patients exhibited a significant enrichment of genes involved in the DNA replication and cell cycle pathways (Fig. 3A and Fig. S4A). Additionally, GCBCs in the inflamed regions of the TI further upregulated mitotic gene expression (Fig. 3B and C, Fig. S4A). Conversely, genes in the unfolded protein response and MHC-II antigen presentation pathways were enriched in GCBCs from non-IBD patients or from the non-inflamed regions in the TI of CD patients (Fig. S4A). Consistent with these gene expression dynamics, we observed a significant increase in the proportions of cluster 1 DZ GCBCs in the inflamed regions, coupled with a significant decrease in the proportions of cluster 0 LZ GCBCs (Fig. 3D). These findings suggest a remodeling of DZ and LZ GC B cell proportions in response to CD in the TI.

In contrast to GCBCs, PCs isolated from CD patients, especially those residing in the inflamed regions of the TI, increased the expression of genes involved in antigen processing and presentation pathway (Fig. 3E and Fig. S4B). Additionally, BCR-related genes were significantly enriched in PCs from the inflamed regions compared to non-inflamed regions within the TI (Fig. S4B). These findings suggested that PCs may serve as antigen-presenting cells in the inflamed regions in the TI of CD patients, consistent with observations in inflamed colons of UC and celiac disease patients [8,30]. Of note, genes associated with endoplasmic reticulum (ER) stress and oxidative phosphorylation were also significantly enriched in PCs from the inflamed regions of the TI (Fig. 3F and Fig. S4B), likely reflecting a specific transcriptional and metabolic state that supports increased antibody production in response to the inflammation in CD [31,32]. In addition, CXCR4, a vital chemokine receptor for PC homing and survival [33], was significantly upregulated in PCs from the inflamed regions of the TI (Fig. 3G). It has been shown that CXCR4-expressing PCs are positively associated with human colon inflammation [34]. We then measured CXCR4 expression in the TI of CD patients (Table S1, validation cohort I) using flow cytometry and confirmed that CXCR4 expression was positively correlated with disease severity (Fig. 3H).

The analysis of gene expression in MBCs discovered that interferon (IFN) response-related pathways were significantly enriched in cells from the inflamed regions relative to the non-inflamed regions in the TI (Fig. S4C). We then calculated the expression of both type I and type II IFN response pathways in individual cell subsets. While the CD inflammation did not significantly alter the expression of IFN response genes in GCBCs, we found that MBCs and PCs from CD patients significantly increased the expression of these genes (Fig. 3I). Moreover, MBCs from the inflamed regions in the TI further increased the expression of IFN response genes. Of note, the expression of IFN response genes was comparable between PCs isolated from the inflamed and non-inflamed regions in the TI of CD patients (Fig. 3I and Fig. S4D and E).

To explore regulatory networks driving the observed transcriptional changes after CD, we utilized the single-cell regulatory network inference and clustering (SCENIC) framework [35] to compare the activities of transcription factor-associated gene networks, or regulons, across different B cell subsets and between CD patients and non-IBD individuals (Table S5). For instance, we found that the activities of regulons associated with mitosis, such as E2F2 and E2F7 that were enriched in DZ (cluster 3) GCBCs (Fig. S5A), were elevated in GCBCs in the TI of CD patients, especially in the inflamed regions (Fig. S5A). This observation was consistent with the result of differential gene expression analysis (Fig. 3A–C and Fig. S4A) and the increased proportion of DZ GCBCs (Fig. 3D). Similarly, regulons associated with mitosis were also enriched in cluster 2 PCs, which exhibited the highest cell-cycle signature score (Fig. S2G), and their activity was increased in the blood and colons in CD patients (Fig. S5B). Among regulons in the MBCs, we observed that resident-like (cluster 2) MBCs upregulated regulons associated with immune cell activation, such as JUN, JUND and KLF6 (Fig. S5C). Moreover, the activities of these regulons in MBCs were increased in CD patients compared with non-IBD patients, implying the potential functions of these MBCs in CD. Together, these findings reveal that CD induces distinct sets of transcriptional programs that could confer different functions and fates in various B cell subsets.

### Circulating PCs and resident-like MBCs are positively associated with disease activity in CD patients

**Figure 4.**
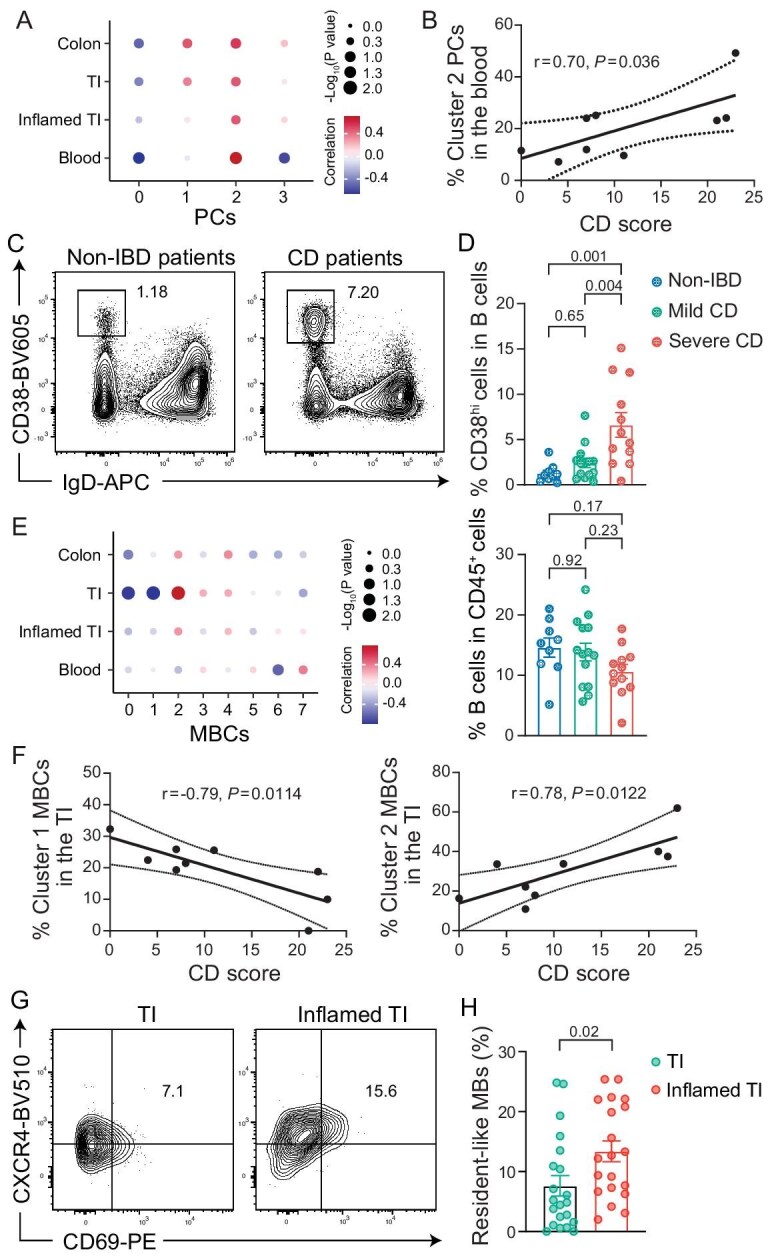
Disruptions in PC and MBC composition associated with disease activity in CD patients. (A) Dot plot showing the correlation scores between disease scores and percentage of each PC cluster (column) in each tissue (row) in CD patients. (B) Linear regression with 95% confidence bands showing the correlation between disease scores and percentages of cluster 2 PCs in the blood of CD patients (*n* = 9). (C) Representative flow plots showing the percentages of CD38^hi^IgD^–^ cells in total B cells from the blood of non-IBD (left) or CD (right) patients. (D) Quantification of the percentages of CD38^hi^ cells in total B cells (left) or B cells in total immune cells (right) in the blood of non-IBD patients (blue, *n* = 9) and mild (green, *n* = 14) or severe (red, *n* = 12) CD patients. Bars display the mean ± s.e.m. (E) Dot plot showing the correlation scores between disease scores and percentages of each MBC cluster (column) in each tissue (row) in CD patients. (F) Linear regression with 95% confidence bands showing the correlation between CD scores and percentages of cluster 1 (left) or cluster 2 (right) MBCs in the TI of CD patients (*n* = 9). (G and H) Representative flow plots (G) and quantification (H) showing the percentages of gut resident-like MBCs (CXCR4^+^CD69^+^) in total MBCs from the inflamed (right) or non-inflamed (left) regions of CD patients. Bars display the mean ± s.e.m. *P* values were calculated using the Pearson test (A, B, E, F), one-way ANOVA with Tukey's multiple-comparison test (D) or two-tailed t-test (H).

We next analyzed the proportions of MBCs and PCs in different tissues of CD and non-IBD patients (Fig. S6A). In the colons, the proportions of MBCs tended to be higher in CD patients compared to non-IBD donors, while the proportions of PCs in CD patients showed a slight decrease. Conversely, in the blood, the proportions of MBCs and PCs exhibited opposite changes compared to those in the colon (Fig. S6A). Of note, the overall proportions of most individual subclusters of PCs and MBCs in CD patients and non-IBD donors were similar (Fig. S6B and C). To further assess the impact of the inflammation on the abundances of each cell subset in CD patients, we analyzed the correlations between individual cell proportions and disease severity as reflected by SES-CD scores (Fig. 4A and Fig. S6D). The analysis revealed that the proportion of PCs in the blood (Fig. S6D), particularly cluster 2 PCs with high expression of mitotic genes (Fig. S2G), was positively correlated with disease severity (Fig. 4A and B, Fig. S6E). We validated this finding using multi-parameter flow cytometry (Fig. S6F) in a new cohort of patients (Table S1, validation cohort II). The frequencies of PCs (CD3^–^CD19^+^CD38^hi^IgD^–^) in the blood, which were mainly KI67-expressing cycling cells (Fig. S6G), were moderately increased in mild CD patients and significantly increased in severe CD patients compared to non-IBD individuals (Fig. 4C and D). In correlation analysis for MBCs, we found that the proportion of total MBCs did not exhibit a significant correlation with disease severity (Fig. S6D). However, when examining individual MBC subclusters, the proportion of cluster 1 MBCs in the non-inflamed TI regions was negatively correlated with disease severity (Fig. 4E and F, Fig. S6H). In contrast, the proportion of cluster 2 MBCs, which exhibited resident-like phenotype (Fig. 2B), in the non-inflamed TI regions, and to a lesser extent in the colon and inflamed TI regions, was significantly positively correlated with disease severity (Fig. 4E and F). We further confirmed that the percentages of resident-like MBCs were significantly elevated in the inflamed regions of the TI in an independent cohort of patients by flow cytometry (Table S1, validation cohort III) (Fig. 4G and H). Taken together, these findings suggest that PCs in the circulation and resident-like MBCs in the TI positively contribute to inflammation in CD patients.

### Distinct CSR patterns between MBCs and PCs in response to CD

**Figure 5.**
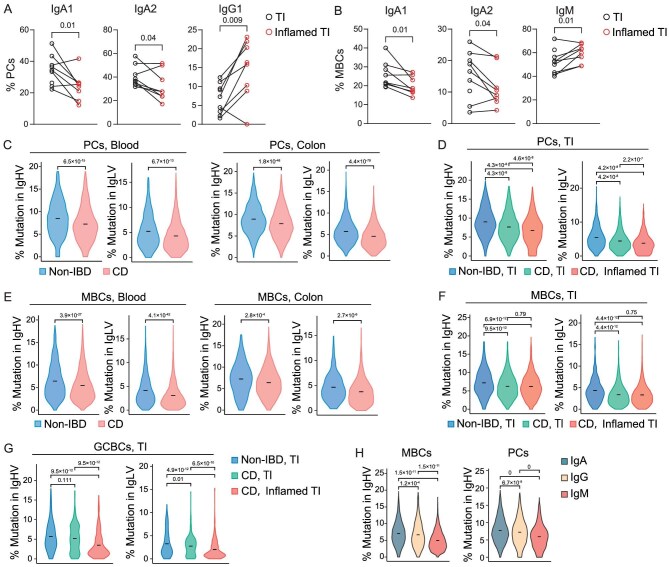
Disruptions of Ig class switch recombination and somatic hypermutation in CD patients. (A and B) Quantification of the frequencies of indicated isotype-expressing cells in PCs (A) and MBCs (B) in the visibly healthy (black) or inflamed (red) TI regions in individual CD patients, connected by lines (*n* = 8). (C and D) Violin plot showing the distribution of Ig mutation rates in PCs in the blood and colon of non-IBD (blue) and CD (red) patients (C), or in the TI of non-IBD patients (blue) and the visibly healthy (green) or inflamed (red) TI regions of CD patients (D). Crossbars display the means. (E and F) Violin plot showing the distribution of Ig mutation rates in MBCs in the blood and colon of non-IBD (blue) and CD (red) patients (E), or in the TI of non-IBD patients (blue) and the visibly healthy (green) or inflamed (red) TI regions of CD patients (F). Crossbars display the means. (G) Violin plot showing the distribution of Ig mutation rates in GCBCs in the TI of non-IBD patients (blue) and the visibly healthy (green) or inflamed (red) TI regions of CD patients. Crossbars display the means. (H) Violin plot showing the distribution of Ig heavy chain variable genes in IgA^+^, IgG^+^ or IgM^+^ MBCs (left) or PCs (right) in CD patients. Crossbars display the means. *P* values were calculated using two-tailed paired t-test (A, B), two-tailed t-test (C, E) or one-way ANOVA with Tukey's multiple-comparison test (D, F, G, H).

Previous studies have shown that PCs in UC patients are skewed from IgA toward IgG1 with the most pronounced difference in the inflamed colon samples. While there was a trend of the skew from IgA toward IgG in PCs in the non-inflamed colonic regions of CD patients, the overall distributions of each Ig isotype in PCs and GCBCs were comparable between CD patients and non-IBD donors (Fig. S7A and B). Within the TI of CD patients, we observed a significant decrease in IgA PCs and a marked increase of IgG, particularly IgG1 PCs in the inflamed regions compared to non-inflamed regions (Fig. 5A and Fig. S7C). The analysis of isotype distributions in MBCs also revealed a slight reduction of IgA in CD patients compared to non-IBD patients (Fig. S7D). Furthermore, we observed a reduction of IgA MBCs in the inflamed regions compared to non-inflamed regions in the TI of CD patients (Fig. 5B). In contrast to IgG PCs, IgG MBC proportions in the inflamed and non-inflamed TI regions were comparable (Fig. S7E). Instead, IgM MBCs were increased in the inflamed TI regions (Fig. 5B). Therefore, in contrast to the skew from IgA toward IgG in PCs, the CD inflammation appears to selectively impair the CSR of IgA in MBCs, leading to the increase of IgM-expressing cells.

### Somatic hypermutation is impaired in CD patients

We observed that PCs in CD patients exhibited reduced somatic hypermutations (SHMs) in both heavy and light chains in the blood, colon and TI, particularly in the inflamed regions (Fig. 5C and D, Fig. S8A and B). This finding is consistent with previous studies reporting decreased SHMs in PCs in the inflamed regions of the colon in patients with UC [8,9]. Furthermore, we observed a similar decline pattern of SHMs in MBCs and GCBCs in CD patients (Fig. 5E–G and Fig. S8C–E). The reduction of SHMs in CD patients was independent of isotype (Fig. S8F–H). We also measured the changes in SHM levels across different regions in the TI and found that the increases of Ig mutations from MBCs to PCs were smaller in the inflamed regions than in the non-inflamed regions, indicating that the SHM after MBC reactivation in inflamed regions was impaired (Fig. S8I). Furthermore, in non-IBD subjects, we found that the number of SHMs was comparable in IgA^+^ and IgG^+^ cells, and significantly higher than that of IgM^+^ cells in MBCs and PCs (Fig. S8J). However, in CD patients, the numbers of SHMs in IgG^+^ cells were significantly lower than that of IgA^+^ cells (Fig. 5H). Therefore, the process of SHMs, particularly in IgG^+^ cells, and potentially the associated antibody affinity maturation, are significantly impaired in CD patients.

### Rewired memory B cell responses in CD patients

**Figure 6.**
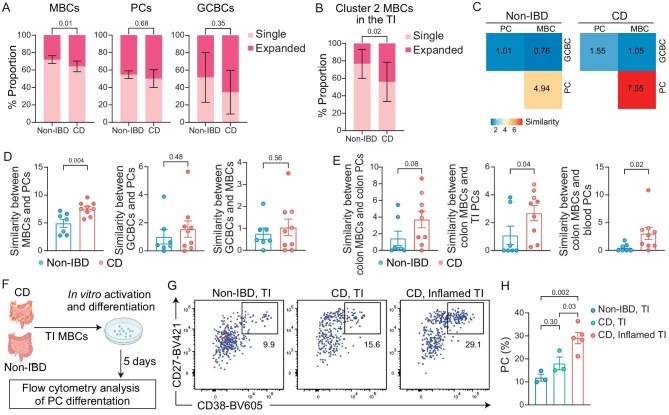
Dysregulated MBC repertoire in CD patients. (A) Quantification of the proportions of cells carrying single or expanded Ig clonotypes in MBCs, PCs and GCBCs from the TI of non-IBD (*n* = 7) and CD (*n* = 9) patients. Bars display the mean ± s.e.m. (B) Quantification of the proportions of cells carrying single or expanded Ig clonotypes in cluster 2 MBCs from the TI of non-IBD (*n* = 7) and CD (*n* = 9) patients. Bars display the mean ± s.e.m. (C) Heatmap showing the Ig repertoire similarities (see ‘Methods’) between the indicated cell types in non-IBD (left) and CD (right) patients. (D) Quantification of Ig similarities between indicated cell types in non-IBD (blue, *n* = 7) and CD (red, *n* = 9) patients. Bars display the mean ± s.e.m. (E) Quantification of Ig similarities between colonic MBCs and colonic PCs (left), or colonic MBCs and PCs in the TI (middle) or blood (right) from non-IBD (blue, *n* = 7) and CD (red, *n* = 9) patients. Bars display the mean ± s.e.m. (F) A schematic diagram of the isolation and culture of intestinal MBCs. (G and H) Representative flow plots (G) and quantification (H) showing the percentages of PCs (CD27^+^CD38^hi^) in cultured cells originally isolated from the TI of non-IBD patients (left), the visibly healthy (middle) or inflamed (right) TI regions of CD patients. Bars display the mean ± s.e.m (H). *P* values were calculated using Mann–Whitney test (A, B), two-tailed t-tests (D, E) or one-way ANOVA with Tukey's multiple-comparison test (H).

We next quantified the usage frequencies of IgHV genes across different B cell subsets in non-IBD and CD patients (Fig. S9A). We observed that MBCs exhibited the most pronounced disparities in IgHV gene usage, with minimal distinctions observed in GCBCs and PCs. For example, the usage frequency of IGHV3-23, which recognizes the gut microbiome [36], was specifically increased in MBCs in CD patients. To further determine the impact of CD on the BCR repertoire, we examined the diversity of the Ig sequences in CD and non-IBD patients using Shannon entropy scores (see ‘Methods’). We found that the overall clonal diversity in three major antigen-experienced B cell populations was similar between CD and non-IBD patients (Fig. S9B). Additionally, there was no significant difference in the clonal diversity between cells from non-inflamed and inflamed regions of the TI in CD patients (Fig. S9C). We then investigated the clonal expansion of different B cell populations in non-IBD and CD patients (see ‘Methods’). The clonal expansion between non-inflamed and inflamed regions of the TI in CD patients was comparable (Fig. S9D). However, there was a significant increase in the proportion of cells harboring expanded BCR clonotypes in MBCs (Fig. 6A and Fig. S9E), particularly in resident-like (cluster 2) MBCs in the TI (Fig. 6B and Fig. S9F), in CD patients compared to non-IBD patients. Conversely, the clonal expansion of GCBCs and PCs was comparable in CD and non-IBD patients (Fig. 6A and Fig. S9F). Together, these findings suggest that chronic inflammation leads to the reactivation and expansion of MBCs in CD patients.

Next, we analyzed the similarity of IgHV clonotypes (see ‘Methods’) between different antigen-experienced B cell populations in CD and non-IBD patients [37]. We found a significant increase in shared clonotypes between MBCs and PCs in CD patients compared to non-IBD patients (Fig. 6C and D). In contrast, the clonotype similarity between GCBCs and PCs or MBCs remained comparable (Fig. 6C and D). Consistently, the clonotype similarity between MBCs and PCs, but not between GCBCs and PCs or MBCs, was increased in the inflamed regions compared to non-inflamed regions of the TI (Fig. S10A). Notably, the increased clonotype similarity with PCs was observed across different MBC clusters in CD patients (Fig. S10B). Moreover, the analysis of MBCs and PCs across different tissues revealed an overall induction in the similarity in CD patients compared with non-IBD individuals (Fig. S10C), reflecting an enhanced migration and differentiation of MBCs and PCs in CD patients. Particularly, the similarities between MBCs in the colon and PCs in the TI, colon or blood were all significantly increased (Fig. 6E and Fig. S10C), suggesting a systemic increase in the contribution of intestinal MBCs to PCs in CD patients. To experimentally determine the impact of CD on the differentiation of intestinal MBCs toward PCs, we cultured MBCs isolated from the TI of a new cohort of CD patients and non-IBD individuals (Table S1, validation cohort IV) (Fig. 6F). We observed that MBCs from CD patients, especially in the inflamed regions, differentiated more rapidly into PCs than MBCs isolated from non-IBD individuals (Fig. 6G and H). Collectively, these findings reveal that chronic inflammation in the intestines facilitates the direct differentiation of MBCs to PCs in CD patients.

## DISCUSSION

Alterations in PC numbers and Ig isotype usage frequencies among IBD patients in comparison to healthy controls were documented decades ago [38,39]. Recent advances in single-cell technologies have further substantiated these findings, illustrating dysregulated B cell responses in IBD patients, marked by an expansion of naïve B cells and IgG^+^ PCs [8,9]. However, these studies are limited to UC patients with a primary focus on the analysis of PCs in the colon. In this study, we recruited CD patients and non-IBD subjects and conducted a comprehensive analysis of the transcriptional state and BCR repertoire of all types of antigen-experienced B cells collected from the TI, colon and blood.

Consistent with the observed shift from IgA to IgG in PCs from UC patients compared with healthy controls [8,9], our study identified a decreased proportion of IgA1^+^ and IgA2^+^ PCs, along with an increase in IgG1^+^ PCs in the inflamed regions of the TI of CD patients. Consistently, IgG PCs were identified in a cellular module associated with inflammation in a subset of CD patients [5]. Notably, PCs in CD patients also upregulated genes related to ER stress, cell division, and MHC-II-mediated antigen presentation pathways, mirroring observations in UC patients [8]. Furthermore, the percentage of PCs in the blood of CD patients positively correlated with disease activity, consistent with findings in UC patients [9]. Finally, reduced SHM of Ig variable genes was observed in CD, similar to UC patients [8,9]. These findings collectively demonstrate that although CD and UC are two distinct IBD subtypes, they share similar alterations in cell composition, transcriptional state, Ig isotype usage and mutations of PCs.

The current understanding of MBC heterogeneity in mice is mainly defined according to their expression patterns of Ig isotypes or several surface markers, such as CD80, PD-L2 and CD73 [40,41]. In humans, the heterogeneity of MBCs is less studied, except the typical (CD21^+^CD27^+^) and atypical (CD21^−^CD27^+^ or CD21^−^CD27^−^CD11c^+^) MBCs that have been reported [19,22]. In our study, we identified MBCs through the coupled analysis of the transcriptional state with antigen-binding-induced BCR modifications (SHM and CSR) for individual cells. This approach, not limited to using CD21 and CD27, allowed us to capture all MBCs *in silico*, including atypical ones, for a comprehensive analysis of their heterogeneity and responses in CD. Our analysis revealed a decrease in IgA^+^ MBC proportions in the inflamed TI of CD patients. Unlike PCs, the proportion of IgG^+^ MBCs remained comparable, whereas IgM^+^ MBCs were increased in CD patients compared to non-IBD subjects, indicating impaired CSR. The increased IgG class switching in UC has been attributed to the expansion of a subset of IFN-imprinted NBCs [9], considering that IFN-γ-mediated type 1 immunity is known to be associated with IgG production [42]. We found that MBCs in CD patients, particularly in the inflamed TI, upregulated genes related to IFN-response pathways. Thus, upon reactivation, IgM^+^ MBCs may favor class-switching to IgG in response to IFN signaling, which contributes to the elevated IgG^+^ PC formation in CD. Notably, while the effect of IFN signaling in NBCs in CD remains to be studied, we did not find changes in the expression of IFN-response pathways in GCBCs, indicating a minimal contribution of IFN signaling in GCBCs to the expansion of IgG^+^ PCs in CD. Moreover, we also observed more significant alterations of IgHV usage frequencies in MBCs than in GCBCs and PCs in CD patients, including IGHV3-23. IGHV3-23 is known for its reactivity against microbiota and is enriched in antibodies targeting malondialdehyde acetaldehyde adducts, which are formed under oxidative stress [43]. In addition, MBCs from CD patients exhibited increased usage of IgHV3-48, which was overrepresented in transglutaminase 2 (TG2)-specific PCs and MBCs from patients with celiac disease [44]. Notably, high levels of anti-TG2 autoantibodies have been detected in the serum of CD patients [45], suggesting potential functions in CD. These analyses reveal that MBCs in CD patients are enriched for dysregulated Ig responses with potential microbiome and autoantigen reactivity.

Our analysis identified a subset (cluster 2) of MBCs that exhibited high expression of CD69 and tissue-resident gene signatures. These cells were enriched in intestinal tissues and virtually absent in the blood, resembling recently proposed gut-resident MBCs [17]. Intriguingly, in CD patients, these resident-like MBCs exhibited increased clonal expansion, and their abundance was positively correlated with the disease activity, indicating their contributions to CD pathogenesis. These resident-like MBCs upregulated multiple regulons associated with immune cell activation, including KLF6. It has been shown that KLF6 expression is upregulated in the intestinal tissue of IBD patients and animal models [46], and its chromatin accessibility in MBCs is reduced by IFN-blocking antibody treatment [47]. Furthermore, KLF6 suppresses BCL6 expression by elevating PRDM1 abundance in macrophages [48]. Thus, future studies will be needed to determine the function of KLF6 in the differentiation and activation of gut resident-like MBCs. Additionally, we observed that resident-like MBCs highly expressed AREG, a member of the epidermal growth factor family that regulates tissue inflammation and repair [49]. AREG has been reported to exacerbate intestinal fibrosis in CD patients [50]. The expression of AREG can be promoted by TNF and Il-1β signaling [51], and genes related to these signaling pathways were upregulated in MBCs in the inflamed regions in the TI. Furthermore, AREG expression has been identified in auto-reactive B cells in rheumatoid arthritis patients [24]. Further studies on the antigen-binding specificity of resident-like MBCs in the intestines and the function of AREG that they secrete may help unveil underlying mechanisms of B cell pathogenesis in CD.

The unique property of Ig sequences allowed us to trace the fate of different B cell populations in humans. We found that IgHV clonotype similarity between PCs and MBCs significantly increased in CD, indicating a biased differentiation toward PCs rather than GCBCs after MBC reactivation under chronic inflammation. In mice, the fate of MBCs upon reactivation has been linked to their heterogeneities. For instance, IgM^+^ or CD80^−^PD-L2^−^ MBCs tend to re-enter the GC while IgG^+^ or CD80^+^PD-L2^+^ MBCs prefer to differentiate into PCs [40,41,52,53]. However, the comparable cluster compositions in MBCs in CD patients and non-IBD controls, as well as the similar increase in clonotype similarity with PCs across different MBC clusters, suggest that the increased direct differentiation to PCs is not due to obvious alterations in MBC heterogeneity in CD. Recent studies in humans demonstrate that the fate of MBCs upon secondary exposure could be influenced by the feedback of pre-existing antibodies [54]. Further studies are needed to determine the signals in the microenvironment, e.g. antibodies resulting from the dysregulated PC responses or inflammatory cytokines, that dictate MBC fate in CD.

In summary, our study provides unprecedented detail into the perturbations of different antigen-experience B cell populations in various tissues in CD, revealing the heterogeneity of MBCs in the intestinal mucosa and the potential roles of gut resident-like MBCs during CD pathogenesis.

## METHODS

The detailed methods and materials are available as Supplementary Data at *NSR* online.

## Supplementary Material

nwaf009_Supplemental_Files
