# Supplementary material for: The multi-organ landscape of B cells highlights dysregulated memory B cell responses in Crohn's disease
Source: Natl Sci Rev. 2025 Jan 10;12(4):nwaf009. doi: 10.1093/nsr/nwaf009 (PMC11951101; doi:10.1093/nsr/nwaf009)
Supplement: nwaf009_Supplemental_Files [file nwaf009_supplemental_files.zip › Chen et al-Supplementary methods and figures.docx]

**The multi-organ landscape of B cells highlights dysregulated memory B cell responses in Crohn’s disease**

Dianyu Chen^1,2,3^, Song Xu^4^, Shuyan Li^5^, Qiuying Wang^1,2,3^, Hui Li^1,2,3^, Danyang He^2,3^, Yan Chen^6,*^, Heping Xu^1,2,3,*^

**Affiliations:**

^1^Westlake Laboratory of Life Sciences and Biomedicine; Hangzhou, Zhejiang, China. ^2^Laboratory of Systems Immunology, School of Medicine, Westlake University; Hangzhou 310024, Zhejiang, China.

^3^Key Laboratory of Growth Regulation and Translational Research of Zhejiang Province, School of Life Sciences, Westlake University; Hangzhou 310024, Zhejiang, China.

^4^Department of Gastroenterology, The Second Affiliated Hospital Zhejiang University School of Medicine; Hangzhou 310000, China

^5^Department of Nursing, The Second Affiliated Hospital, Zhejiang University School of Medicine; Hangzhou, 310009 China

^6^Center for Inflammatory Bowel Diseases, Department of Gastroenterology, The Second Affiliated Hospital, Zhejiang University School of Medicine; Hangzhou, 310009 China

*Correspondence: chenyan72_72@zju.edu.cn (Y.C.); xuheping@westlake.edu.cn (H.X.)

**Supplementary Methods**

**Human samples**

All procedures with human samples in this study were conducted with approval from the Ethics Committee of The Second Affiliated Hospital Zhejiang University and Westlake University (20191217XHP001). Participants were enrolled from five distinct cohorts after obtaining informed consent from all individuals involved (**Table S1**).

The primary cohort for 5’ scRNA-seq comprised nine patients diagnosed with Crohn’s Disease (CD), six individuals with intestinal polyps, and one healthy subject without any history of Inflammatory Bowel Disease (IBD), immune-mediated disorders, or cancer. Intestinal biopsies and blood samples collected from these participants were used for 5’ droplet-based scRNA-seq.

The validation cohort I for validating differential enrichment of MBC subsets across different tissues comprised sixteen CD patients and one healthy individual without any history of IBD, immune-mediated diseases, or cancer. Intestinal biopsies and blood samples collected from these participants were analyzed by flow cytometry.

The validation cohort II for validating the increased frequency of PCs in the blood of CD patients comprised twenty-six CD patients, two individuals with intestinal polyps, and seven healthy subjects without any history of Inflammatory Bowel Disease (IBD), immune-mediated disorders, or cancer. Blood samples collected from these participants were analyzed by flow cytometry.

The validation cohort III for validating the increased frequency of resident-like MBCs in CD patients comprised twenty-two CD patients. Intestinal biopsies collected from these participants were analyzed by flow cytometry.

The validation cohort IV for validating the PC differentiation potential of intestinal MBCs comprised five CD patients, three individuals with chronic gastritis. MBCs were flow-purified from intestinal biopsies of these participants for *in vitro* culture.

**Single-cell isolation**

Single-cell suspensions from collected biopsies were obtained as previously described[1]. Briefly, fresh biopsies were rinsed in 30 ml of ice-cold PBS and allowed to settle. The specimens were then transferred to 10 ml of epithelial cell solution (RPMI1640 [ThermoFisher, Cat# C22400500BT], 100 U/ml penicillin, 100 μg/mL streptomycin, 2% FBS [ThermoFisher, Cat# 10270106]) freshly supplemented with 200 μl of 0.5 M EDTA. Separation of the epithelial layer from the underlying lamina propria was performed for 15 min at 37°C in a rotisserie rack with end-over-end rotation. The tube was then removed and placed on ice immediately for 10 min before shaking vigorously 15 times. The remnant tissue pieces were placed into a large volume of ice-cold PBS to rinse before transferring to 5 ml of enzymatic digestion mix (RPMI1640, 100 U/ml penicillin, 100 μg/mL streptomycin, and 2% FBS), freshly supplemented with 100 μg/ml of Liberase TM (Roche, Cat# 5401127001) and 100 μg/ml of DNase I (Roche, Cat# 10104159001), at 37°C with 120 rpm rotation for 30 min. The enzymatic dissociation was quenched by adding 1 ml of 100% FBS and 20 μl of 0.5 M EDTA and then placed on ice for 5 min. Samples were then filtered through a 40-μm cell strainer into a new 50-ml conical tube, rinsed with PBS to a final volume of 30 ml, and centrifuged at 500g for 5 min at 4°C. Erythrocytes were depleted using ammonium–chloride–potassium (ACK) lysis buffer prior to antibody labeling.

The peripheral blood was diluted with RPMI1640 and gently overlaid on Ficoll-Paque (GE Healthcare, Cat# 17144003). After centrifugation, the phase containing peripheral blood mononuclear cells (PBMCs) was collected and washed two times with RPMI1640.

**Flow cytometry and fluorescence-activated cell sorting (FACS)**

Isolated cells were stained with antibody cocktails in MACS buffer (pH 7.4; PBS plus 2% FBS and 5 mM EDTA) for 30 min at 4°C. Antibodies used to stain are listed in the **Table S3**. Dead cells were excluded with Fixable Viability Dye eFluor 780 (Thermo Fisher, Cat# 65-0865-18). The BD Cytofix/Cytoperm Fixation/Permeabilization Kit (BD Biosciences, Cat# 554714) was used for intracellular staining. Flow cytometry was performed on Cytoflex (Beckman Coulter), Aurora (Cytek Biosciences) or LSRFortessa (BD Biosciences). Human antigen-experienced B cells (CD45^+^CD19^+^IgD^-^ or CD27^+^) and naïve B cells (CD45^+^CD19^+^IgD^+^CD27^–^) were sorted using MA900 (Sony) with a 100-μm chip.

**5’ Droplet-based scRNA-Seq**

Cell hashing was performed by incubating cells with antibody cocktails containing FACS antibodies and TotalSeq Hashtag antibodies (Biolegend) (1:100 final dilution) for 30 min at 4°C. Next, cells were washed twice and sorted into resuspension buffer (PBS plus 0.04% BSA). 19,000-40,000 cells were loaded per channel using the Chromium Next GEM Single Cell 5’ v1.1 kits (10X Genomics). Gene expression, V(D)J, and feature barcoding libraries were generated following the manufacturer’s instruction (10x Genomics). Libraries were pair-end (150 + 150 bp) sequenced on a Novaseq 6000 system (Illumina).

**Human MBC *in vitro* culture**

MBCs were purified and cultured as previously described[2]. Briefly, MBCs (CD45^+^CD19^+^CD38^–^IgD^–^) were flow-sorted from the intestinal single-cell suspension into the culture medium (RPMI 1640, containing 10% FBS, 100 U/ml penicillin, 100 μg/mL streptomycin, and 0.05mM β-Mercaptoethanol [Gibco]), and cultured in the presence of 1 µg/ml ODN2006 (5’-tcgtcgttttgtcgttttgtcgtt-3’, InvivoGen) for 5 days. The proportion of PCs were measured by flow cytometry.

**BCR analysis**

Single-cell V(D)J sequencing data were processed using the Cellranger toolkit (v6.1.1, 10X Genomics) with refdata-cellranger-vdj-GRCh38-alts-ensembl-5.0.0 reference from 10X Genomics. Low-quality contig sequences were removed, and only cells with exactly one high-quality heavy chain and/or one high-quality light chain were selected. The remaining contig sequences were mapped to the germline reference (202207-4) released from the international ImMunoGeneTics information system[3] (IMGT) using IgBlast (v1.18.0). Further V, D, J and C gene annotations were assigned using the R package alakazam (v1.2.0). V, D, J and C gene usage and hypermutation rate were quantified using custom scripts. Donor-specific B cell clones were identified based on the heavy chain of all cells derived from each donor using Change-O[4] (v1.2.0) DefineClones.py utility. Clones were considered as the same clonotype when they had the same IgHV and IgHJ segments as well as the same CDR3 length and more than 70% similarity in the nucleotide sequence of the CDR3 of the heavy chain. The diversity of selected cells was quantified by the Shannon entropy using alphaDiversity function in the alakazam package. Clones containing more than one cell were defined as expanded clones. To compare the clonal repertoire between two samples, we calculated the similarity defined as the size of the intersection divided by the size of the union of the sample sets and scaled by 100.

$Similarity(A, B)=|A{\cup B|}/{\left| A\cap B \right|\times100}$

The value of similarity is between 0 and 100, the closer to 100, the more clonotype is shared between the two samples, and the closer to 0, the less clonotype is shared.

**scRNA-seq data analysis**

Initial processing and gene expression estimation were performed using the Cellranger toolkit (v6.1.1, 10X Genomics) with refdata-gex-GRCh38-2020-A reference from 10X Genomics. The UMI count matrix was converted to Seurat objects using the R package Seurat[5] (v.4.1.1) operated in RStudio (v2023.12.1+402) for samples from each individual. Doublets and low-quality cells were removed based on custom thresholds for the RNA count, gene number and percentage of mitochondrial genes of individual samples. The filtered UMI count matrix was normalized by the total counts for each cell, scaled by 10^6^, and then log-transformed using log1p. The top 2,000 highly variable genes were identified using variance-stabilizing transformation, and genes that were repeatedly variable across datasets were used for batch correction. We identified ‘anchors’ between pairs of datasets, which represented pairwise correspondences between individual cells (one in each dataset). Then, we used these ‘anchors’ to harmonize the datasets and obtained an integrated matrix for downstream analysis. The integrated data were scaled and the highly variable genes were used for principal component analysis (PCA). The first 15 principal components were used for Uniform Manifold Approximation and Projection (UMAP) for visualization and graph-based clustering with a resolution of 0.05. Marker genes for each cluster were identified using model-based analysis of single-cell transcriptomics (MAST) test with individual and tissue origin as covariants. Low-quality PCs and other non-B-cell clusters were excluded from the downstream analysis.

Cells were partitioned into naïve/memory B cells, PCs, and GCBCs compartments based on marker genes of each cluster, and we performed further detailed clustering within each compartment. For GCBCs, we directly re-performed PCA, and the first 9 principal components were used for UMAP visualization and graph-based clustering with a resolution of 0.25. For PCs, we split the dataset based on the tissue origin and re-integrated them to remove batch effects from different tissues. Then, we re-performed PCA, and the first 10 principal components were used for UMAP visualization and graph-based clustering with a resolution of 0.15.

For MBC identification, as they have undergone somatic hypermutation (SHM) and class switch recombination (CSR), we hypothesized that antigen-driven alterations in the BCR could aid in distinguishing MBCs from naïve B cells, which possess germline Ig sequences. Naïve/memory B cells were firstly re-clustered and the resulting cluster 2 showed high expression of *IGHD* while almost no expression of *CD27*. Flow-sorted naïve B cells (IgD^+^CD27^-^) in this cluster were used as control cells to establish thresholds for calling antigen-driven mutations, and the thresholds for the hypermutation rates of heavy and light chains were set to 1% and 3%, respectively. Naïve/memory B cells are first screened based on the mutation rate of the heavy chain, and those with a mutation rate exceeding 1% are considered to have undergone mutation and are identified as MBCs. If the mutation rate of the heavy chain does not exceed this threshold, the mutation rate of the light chain is then considered. A mutation rate exceeding 3% in the light chain is also taken as an indication of mutation. If neither the heavy nor light chain mutation rates surpass their respective thresholds, the occurrence of CSR is evaluated. If CSR has occurred, the cells are also classified as MBCs. MBCs were then subset and re-integrated to remove batch effects from tissues and individuals. PCA was re-performed, and the first 12 principal components were used for UMAP visualization and graph-based clustering with a resolution of 0.25.

**Differential gene analysis and gene set enrichment analysis**

Differential expression testing was performed using the FindMarkers function in the Seurat package (v.4.1.1) based on the MAST test. Gene set enrichment analysis was performed using the R package clusterProfiler[6] (v4.4.4) based on the result of differential gene analysis (**Table S4**).

**Identification of gene regulatory networks**

Gene regulatory network analysis was performed using pyscenic package[7] (v0.12.1). The list of human transcription factors (allTFs_hg38.txt), cisTarget database containing transcription factor motif scores for gene promoters and transcription start sites (hg38_10kbp_up_10kbp_down_full_tx_v10_clust.genes_vs_motifs.rankings.feather), and the motif annotation (motifs-v10nr_clust-nr.hgnc-m0.001-o0.0.tbl) were downloaded from the pyscenic resource website (https://resources.aertslab.org/). The regulatory networks between each transcription factor and putative target genes were inferred based on the expression matrix of each antigen-experienced B cell population using grn function in the pyscenic. The networks were pruned based on the catalog of motifs and their gene association using ctx function. The activity (AUC score) of each transcription factor network (regulon) in each cell was performed using aucell function.

**Gene signature score**

Gene signature scores were calculated as previously reported[8]. Briefly, for each targeted gene set, we chose ten times more genes as a control gene set, which was defined by finding the closest genes in terms of expression level and detection rate, aiming to have a comparable distribution. As the control gene set is tenfold larger, its average expression (log2(counts per million (CPM) + 1)) is analogous to averaging over ten randomly selected gene sets of the same size as the targeted gene set. We then obtained a gene signature score by subtracting the average expression value of the control gene set from the average expression value of the targeted gene set for individual cells. For the heatmap showing the IFN signature score, type I and II signature scores were calculated for the indicated population and then scaled among the same cell type between different tissues.

**Data availability**

The transcriptomic sequencing data have been deposited in the Genome Sequence Archive in the National Genomics Data Center, China National Center for Bioinformation/Beijing Institute of Genomics, Chinese Academy of Sciences are publicly accessible at https://ngdc.cncb.ac.cn/gsa-human (bioProject accession: PRJCA023904).

**Supplementary References**

1. Smillie CS, Biton M, Ordovas-Montanes J *et al.* Intra- and Inter-cellular Rewiring of the Human Colon during Ulcerative Colitis. *Cell* 2019; **178**: 714-30 e22.

2. Weisel NM, Weisel FJ, Farber DL *et al.* Comprehensive analyses of B-cell compartments across the human body reveal novel subsets and a gut-resident memory phenotype. *Blood* 2020; **136**: 2774-85.

3. Manso T, Folch G, Giudicelli V *et al.* IMGT(R) databases, related tools and web resources through three main axes of research and development. *Nucleic Acids Res* 2022; **50**: D1262-D72.

4. Gupta NT, Vander Heiden JA, Uduman M *et al.* Change-O: a toolkit for analyzing large-scale B cell immunoglobulin repertoire sequencing data. *Bioinformatics* 2015; **31**: 3356-8.

5. Hao Y, Hao S, Andersen-Nissen E *et al.* Integrated analysis of multimodal single-cell data. *Cell* 2021; **184**: 3573-87 e29.

6. Wu T, Hu E, Xu S *et al.* clusterProfiler 4.0: A universal enrichment tool for interpreting omics data. *Innovation (Camb)* 2021; **2**: 100141.

7. Van de Sande B, Flerin C, Davie K *et al.* A scalable SCENIC workflow for single-cell gene regulatory network analysis. *Nat Protoc* 2020; **15**: 2247-76.

8. Chen D, Wang Y, Manakkat Vijay GK *et al.* Coupled analysis of transcriptome and BCR mutations reveals role of OXPHOS in affinity maturation. *Nat Immunol* 2021; **22**: 904-13.

**Supplementary figures and figure legends**

**
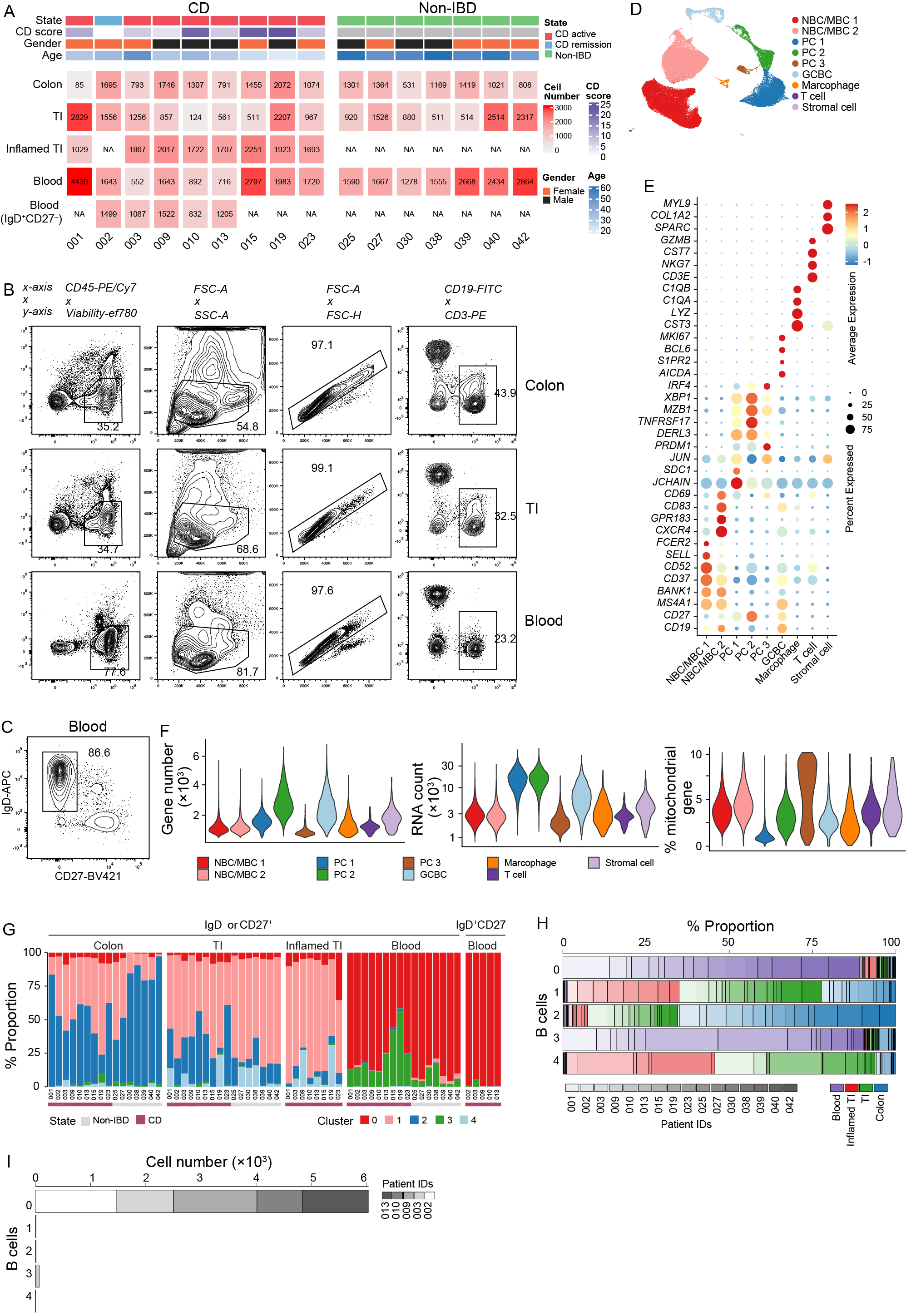
**

**Figure. S1 Characterization of patient samples and B cell subtypes**

**A**, Heatmap showing clinical information and the number of cells, after filtering the low-quality cells (**Supplementary Methods**), purified from the indicated tissues (row) of each CD patient (column). Cells were sorted from the colon, TI, inflamed TI and blood as in **Fig. 1B** (IgD^–^ or CD27^+^) or from the blood as in **Fig. S1C** (IgD^+^CD27^–^).

**B**, Representative flow plots showing the gating strategies for B cells in the indicated tissues.

**C**, Representative flow plot showing the gating strategy for IgD^+^CD27^–^cells in CD19^+^ cells (B) in the blood.

**D**, UMAP visualization of all cell clusters identified in our single cell RNA-seq dataset. Cells are colored by cluster membership.

**E**, Representative marker genes (rows) across cell clusters (columns) in (D). The fraction of cells in the clusters expressed a gene (dot size) and the Z score of mean expression (log_2_(TP10K+1)) of this gene in the cluster.

**F**, Violin plots showing the distribution of gene number (left), RNA count (middle) and percentage of mitochondrial genes (right) in individual clusters in (D).

**G**, The distribution of major B cell clusters (**Fig. 1C**) among tissues of each patient. Cells sorted with different gating strategies were displayed separately.

**H**, The proportion of cells in each major B cell cluster that are purified from each colon (blue), TI (green), inflamed TI (red), or blood (purple) sample via FACS gated as IgD^–^ or CD27^+^.

**I**, The number of cells in each major B cell cluster that are purified from each individual via FACS gated as IgD^+^CD27^–^.

**
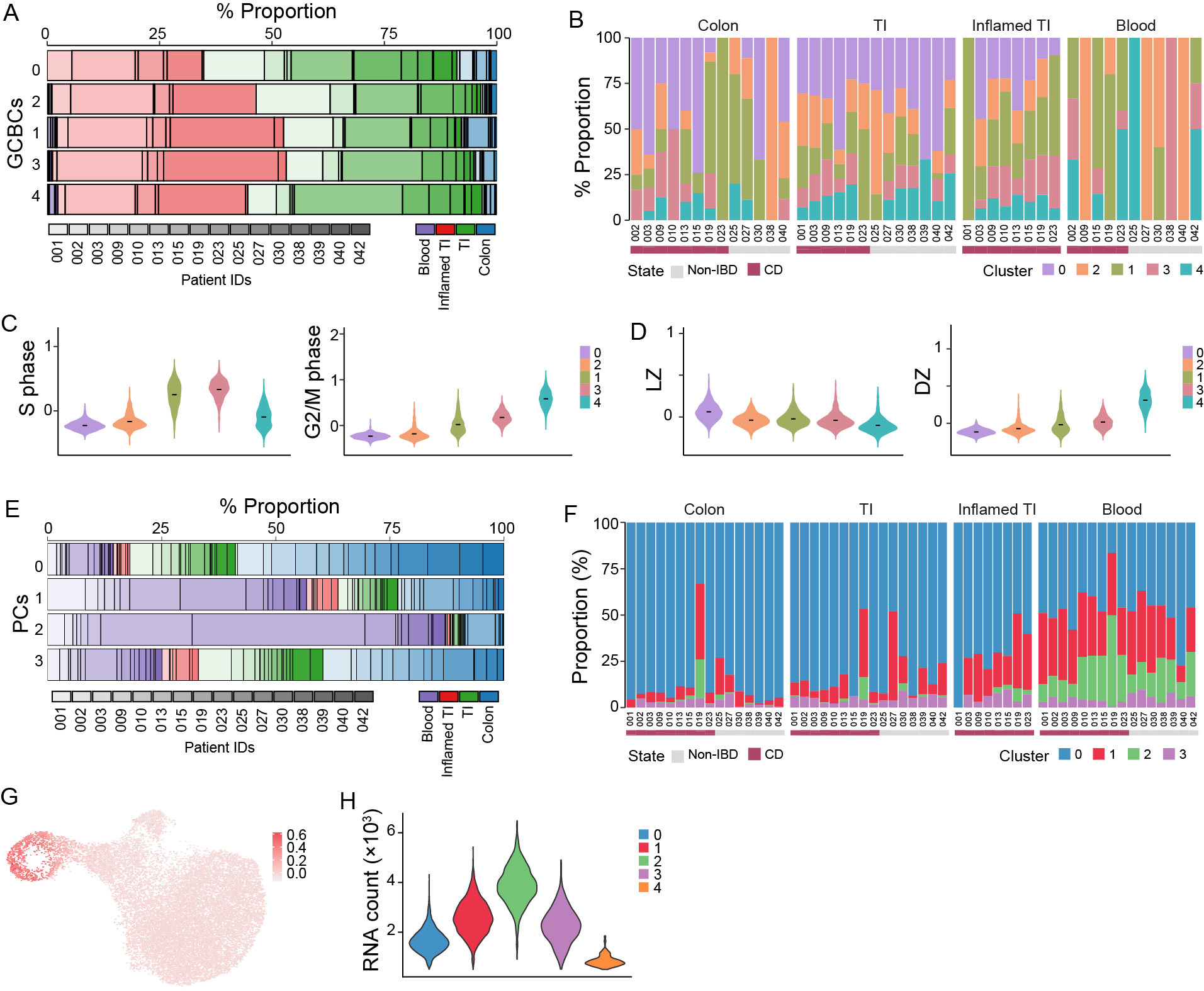
**

**Figure. S2 Proportion and mitotic state of GCBCs and PCs**

**A**, The proportion of cells in each GCBC cluster (**Fig. 1D**) that are purified from each colon (blue), TI (green), inflamed TI (red), or blood (purple) sample via FACS gated as IgD^–^ or CD27^+^.

**B**, The distribution of GCBC clusters among tissues in each individual.

**C**, Violin plot showing the distribution of S (left) or G2/M (right) phase signature scores across GCBC clusters. Crossbars display the means.

**D**, Violin plot showing the distribution of light zone (LZ, left) or dark zone (DZ, right) signature scores across GCBC clusters. Crossbars display the means.

**E**, The proportion of cells in each PC cluster (**Fig. 1F**) that are purified from each colon (blue), TI (green), inflamed TI (red), or blood (purple) sample via FACS gated as IgD^–^ or CD27^+^.

**F**, The distribution of PC clusters among tissues in each individual.

**G**, UMAP plot as in **Fig. 1F** showing the expression of mitotic gene signature in PCs.

**H**, Violin plot showing the distribution of RNA count in each PC cluster.

**
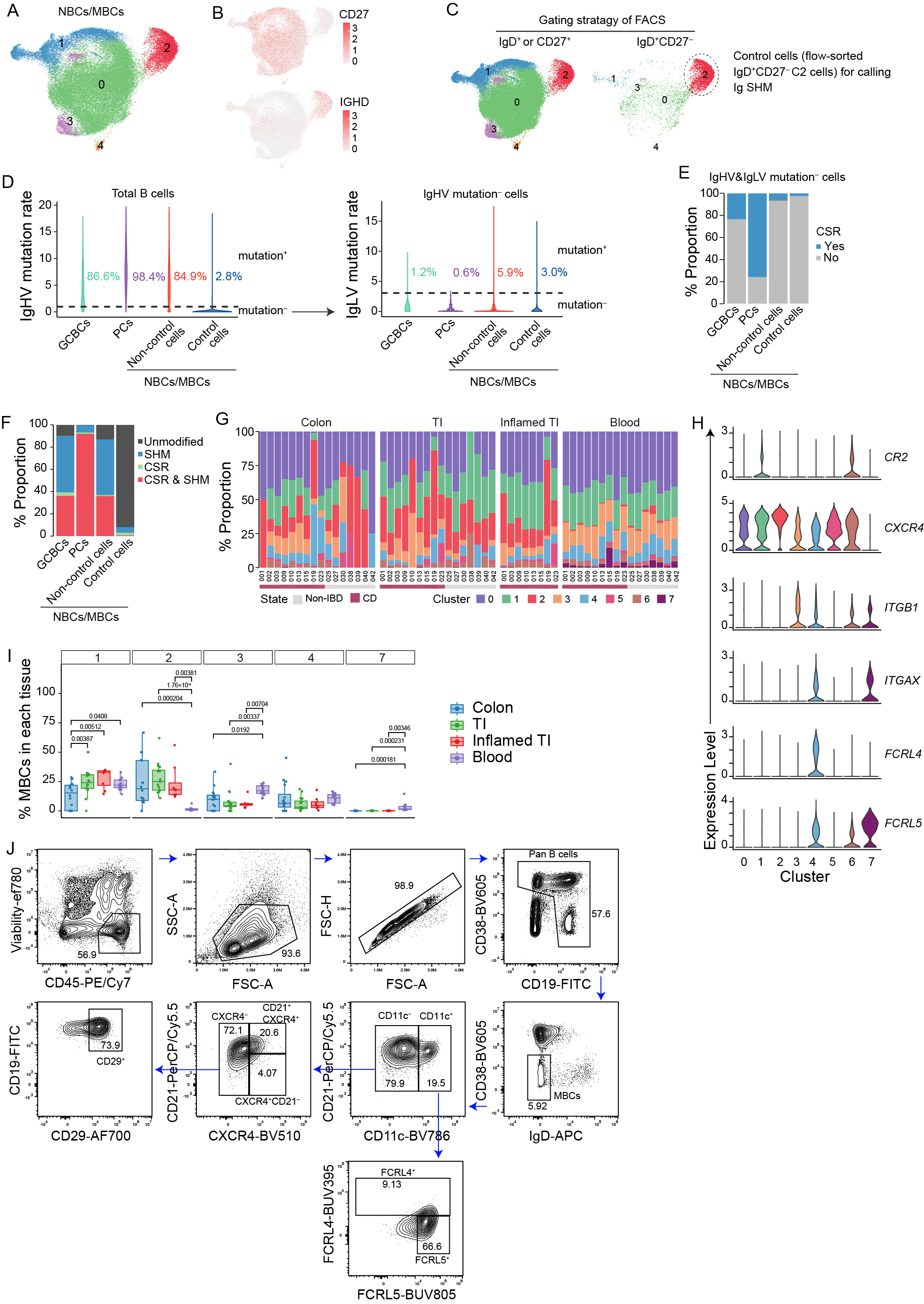
**

**Figure. S3 Characterization and flow cytometry analysis of MBCs.**

**A**, UMAP visualization of naïve/memory B cells (NBCs/MBCs). Cells are colored and numbered by cluster membership.

**B**, UMAP plots as in (A**)** showing the relative expression of *CD27* (top) and *IGHD* (bottom).

**C**, UMAP plots as in (A**)** showing cells purified via FACS gated as IgD^–^ or CD27^+^ (left), or IgD^+^CD27^–^(right). Cluster 2 cells sorted through the IgD^+^CD27^–^ gating (dashed circle) were used as control cells for determining thresholds (D) for calling somatic hypermutation.

**D**, Violin plots showing the mutation rate in the heavy chain of all B cells in our dataset (left), and the mutation rate in the light chain of B cells without mutation in their heavy chain (right). Lines represent 1% and 3% as the threshold for calling mutation in the heavy and light chains. Numbers display the percentage of cells with higher mutation rates than the threshold. Controls cells are cluster 2 cells as defined in (C**)**, and non-control cells are all NBCs/MBCs except control cells.

**E**, The proportion of class-switched (blue) and unswitched (grey) cells in each B cell subset without mutations on both heavy and light chains.

**F**, The proportion of cells carrying SHM and/or CSR within each B cell subset. SHM: cells with Ig SHM but not CSR. CSR: cells with Ig CSR but not SHM. CSR & SHM: cells with both Ig SHM and CSR. Unmodified: cells with neither Ig SHM nor CSR.

**G**, The distribution of MBC clusters among tissues of each patient.

**H**, The relative expression of selected genes across MBC clusters.

**I**, Quantification of the percentage of cells in the indicated clusters in total MBCs in each tissue. P values were calculated using one-way ANOVA with Tukey’s multiple-comparison test.

**J**, Representative flow plots showing the gating strategy for different MBC subsets.

**
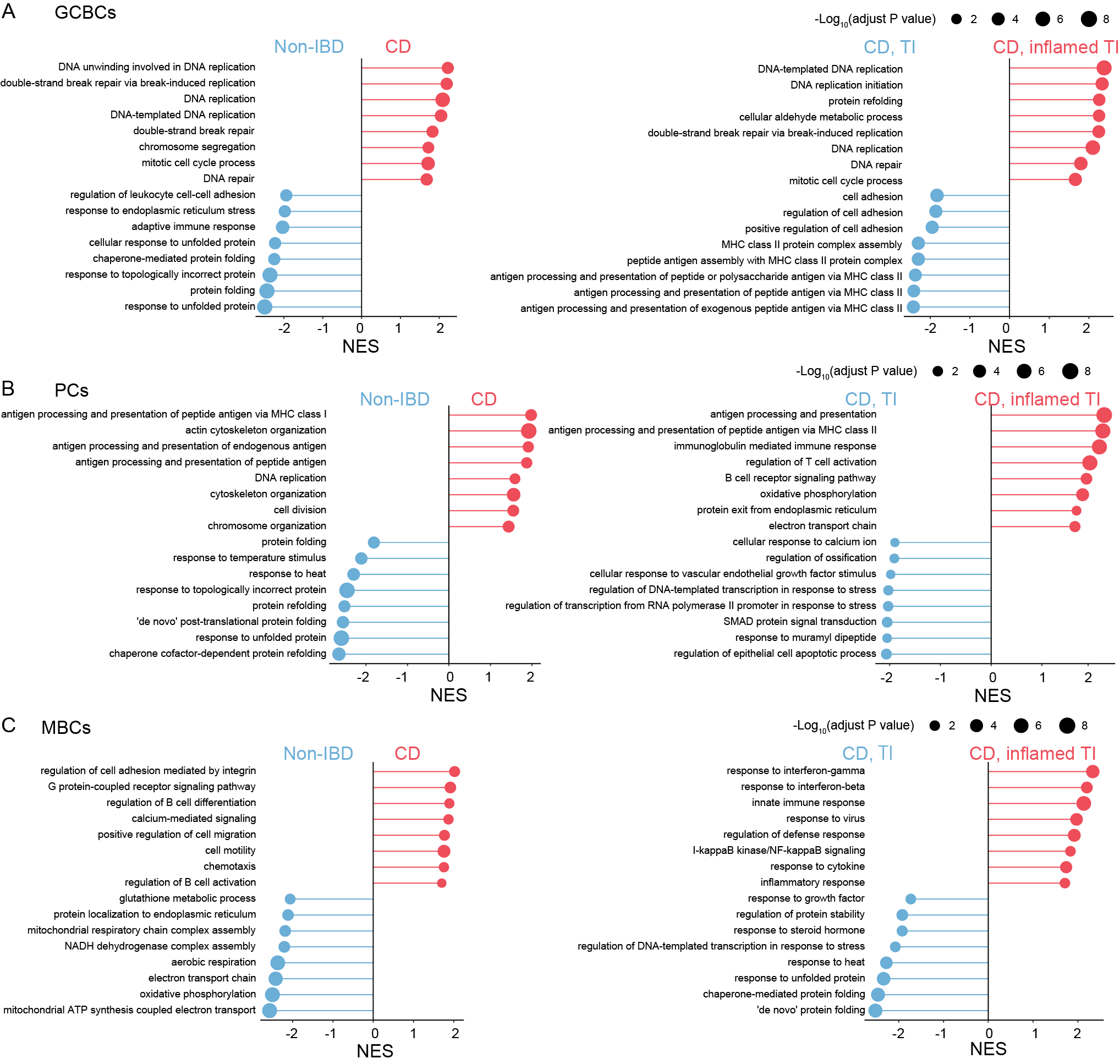
**

**Figure. S4 Significantly enriched pathways in GCBCs, PCs and MBCs in CD patients.**

**A-C**, Differential pathway enriched in GCBCs (A), PCs (B) and MBCs (C) from CD (blue) versus non-IBD (red) patients (left), or from visibly healthy (blue) versus inflamed (red) TIs in CD patients, as evaluated using GSEA.

**
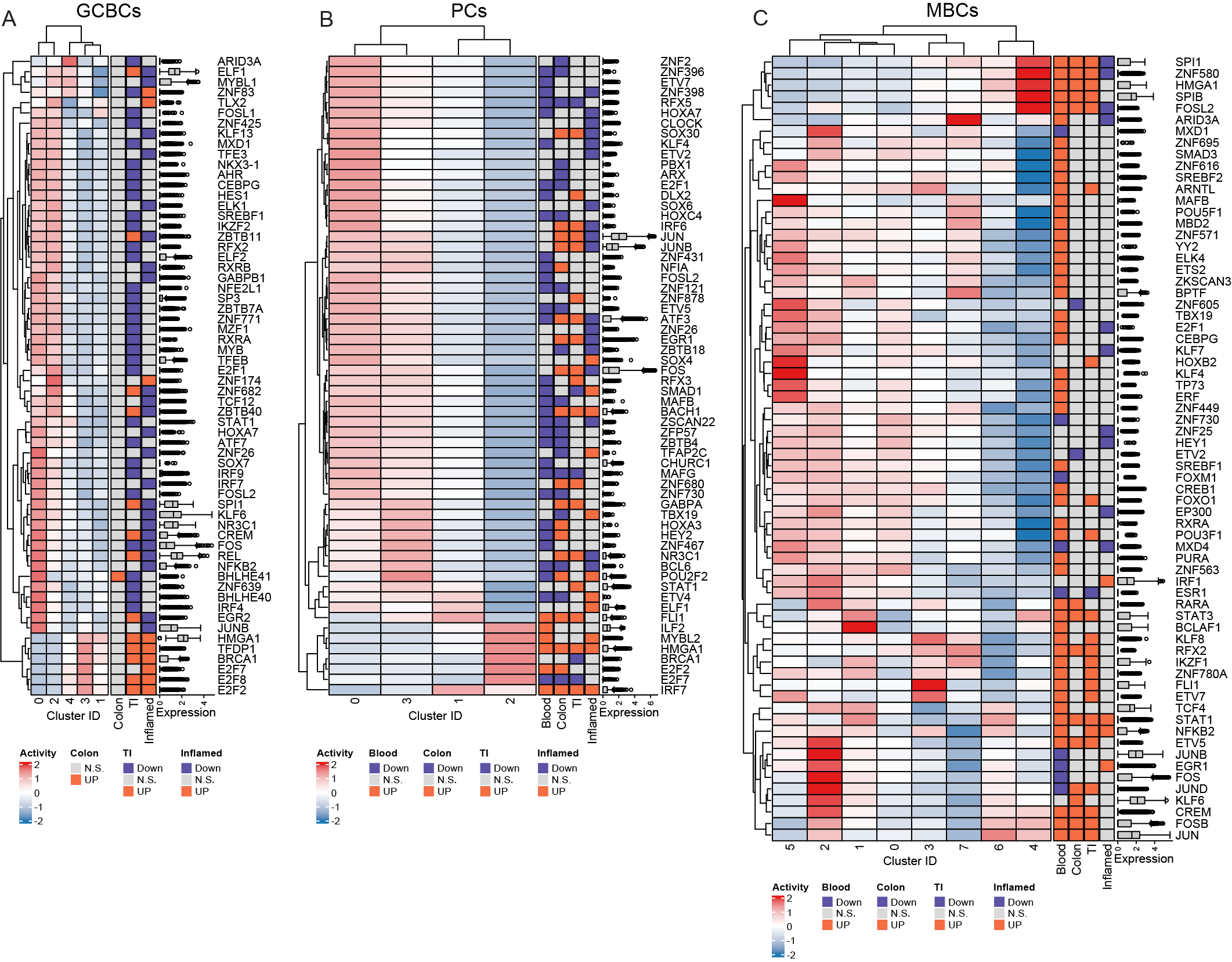
**

**Figure. S5 Altered regulon activity in B cells in CD patients**

**A-C**, Heatmap showing the relative activity (row-wise Z score of AUC score) of each regulon (row, **Supplementary Methods**) in each cluster (column) of GCBCs (A), PCs (B) and MBCs (C). Lateral heatmap showing the difference of regulon activity in each cell population from the colon (Colon), TI (TI) or blood (Blood) between CD and non-IBD patients, or between inflamed and non-inflamed TI regions of CD patients. Boxplots showing the expression abundance (log_2_(TP10K+1)) of indicated transcriptional factors. UP, increased activity in CD versus non-IBD patients or inflamed versus non-inflamed TI regions; Down, decreased activity in CD versus non-IBD patients or inflamed versus non-inflamed TI regions; N.S., no significant difference.

**
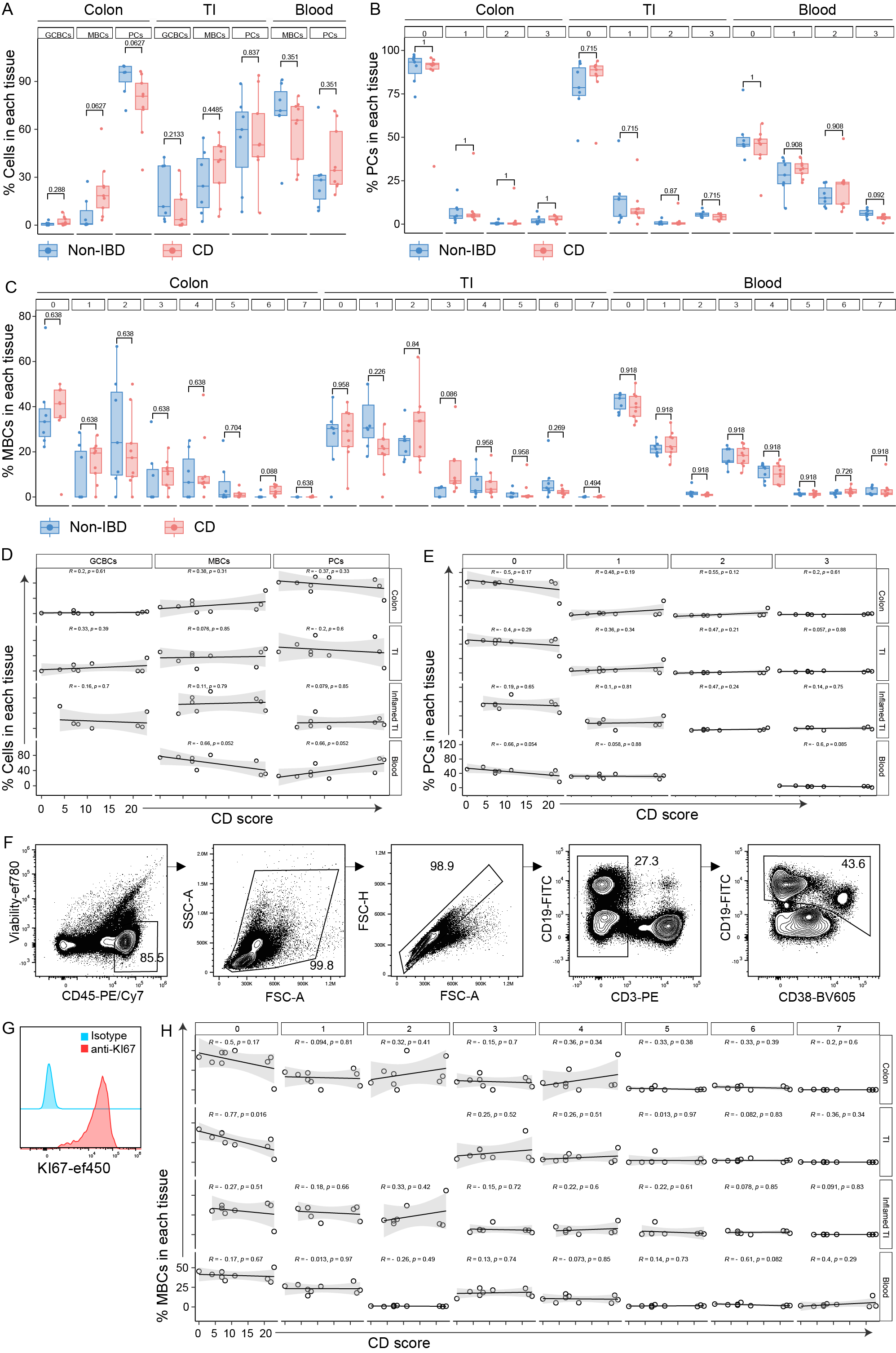
**

**Figure. S6 B cell composition changes associated with CD**

**A**, Quantification of the percentages of indicated cell types among total cells in individual tissues of non-IBD (blue) and CD (red) patients.

**B**, Quantification of the percentages of PCs in indicated clusters among total PCs in individual tissues of non-IBD (blue) and CD (red) patients.

**C**, Quantification of the percentages of MBC in the indicated clusters among total MBCs in individual tissues of non-IBD (blue) and CD (red) patients.

**D**, Linear regression with 95% confidence bands showing the correlation between disease scores and the percentages of each cell type (column) among total cells in each tissue (row).

**E**, Linear regression with 95% confidence bands showing the correlation between disease scores and percentages of each PC clusters (column) among total PCs in each tissue (row).

**F**, Representative flow plots showing the gating strategy for total blood B cells.

**G**, Representative plot showing the expression of KI67 in blood PCs.

**H**, Linear regression with 95% confidence bands showing the correlation between disease scores and percentages of each MBC cluster (column) among total MBCs in each tissue (row).

*P* values were calculated using Mann-Whitney tests and BH-corrected within each tissue (A,B,C) or Pearson test (D,E,H).

**
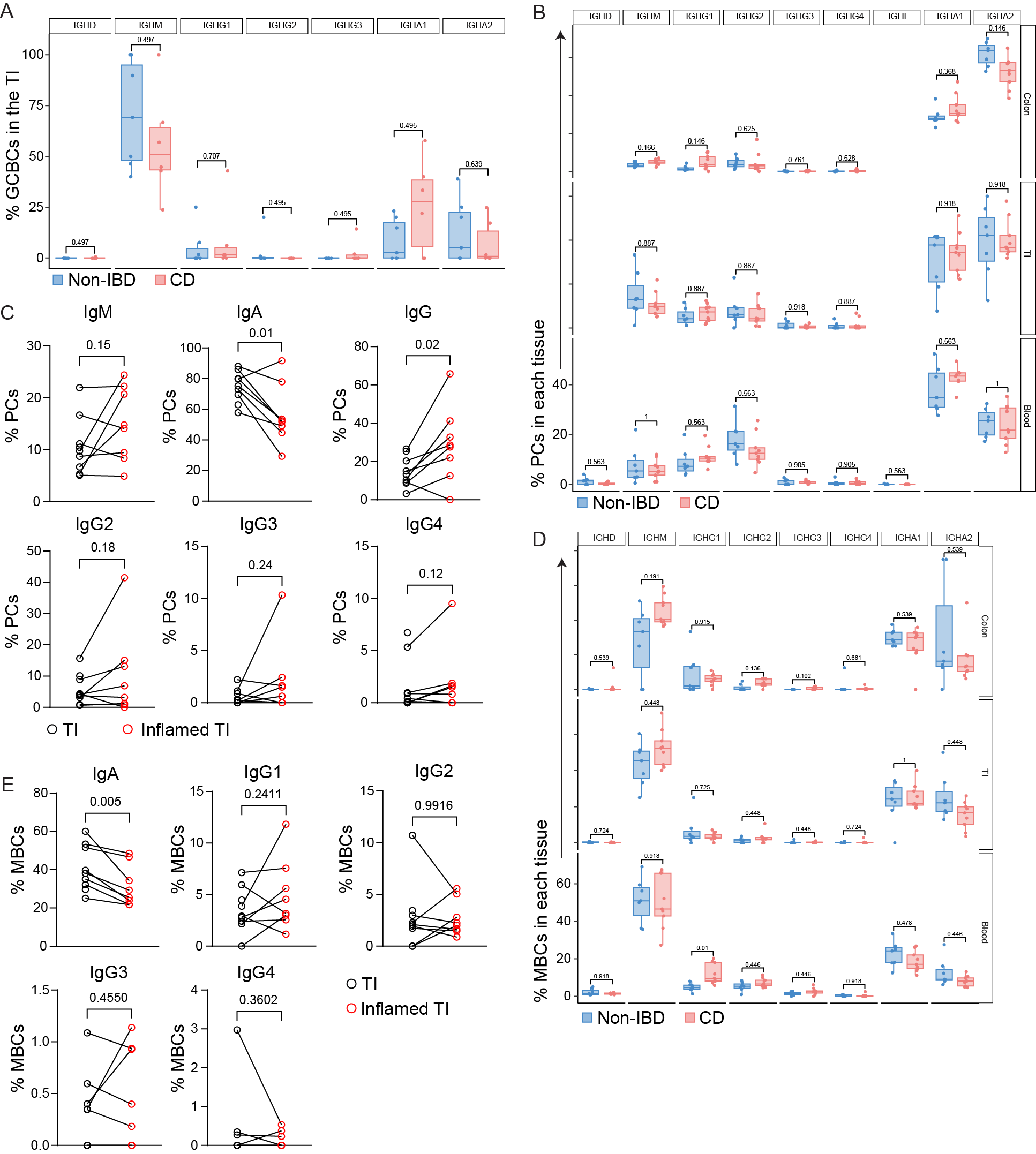
**

**Figure. S7 Dysregulated Ig class switch recombination in CD patients.**

**A**, Quantification of the percentages of each IgH isotype (column) in GCBCs in the TI from non-IBD (blue) or CD (red) patients.

**B**, Quantification of the percentages of each IgH isotype (column) in PCs in each tissue (row) from non-IBD (blue) or CD (red) patients.

**C**, Quantification of the percentages of the indicated IgH isotypes in PCs in the visibly healthy (black) or inflamed (red) TI regions in individual CD patients, connected by lines (n = 8).

**D**, Quantification of the percentages of each IgH isotype (column) in MBCs in each tissue (row) from non-IBD (blue) and CD (red) patients.

**E**, Quantification of the percentages of the indicated IgH isotypes in MBCs in the visibly healthy (black) or inflamed (red) TI regions in individual CD patients, connected by lines (n = 8).

*P* values were calculated using Mann-Whitney tests and BH-corrected within each tissue (A,B,D), two-tailed paired t-test (C,E).

**
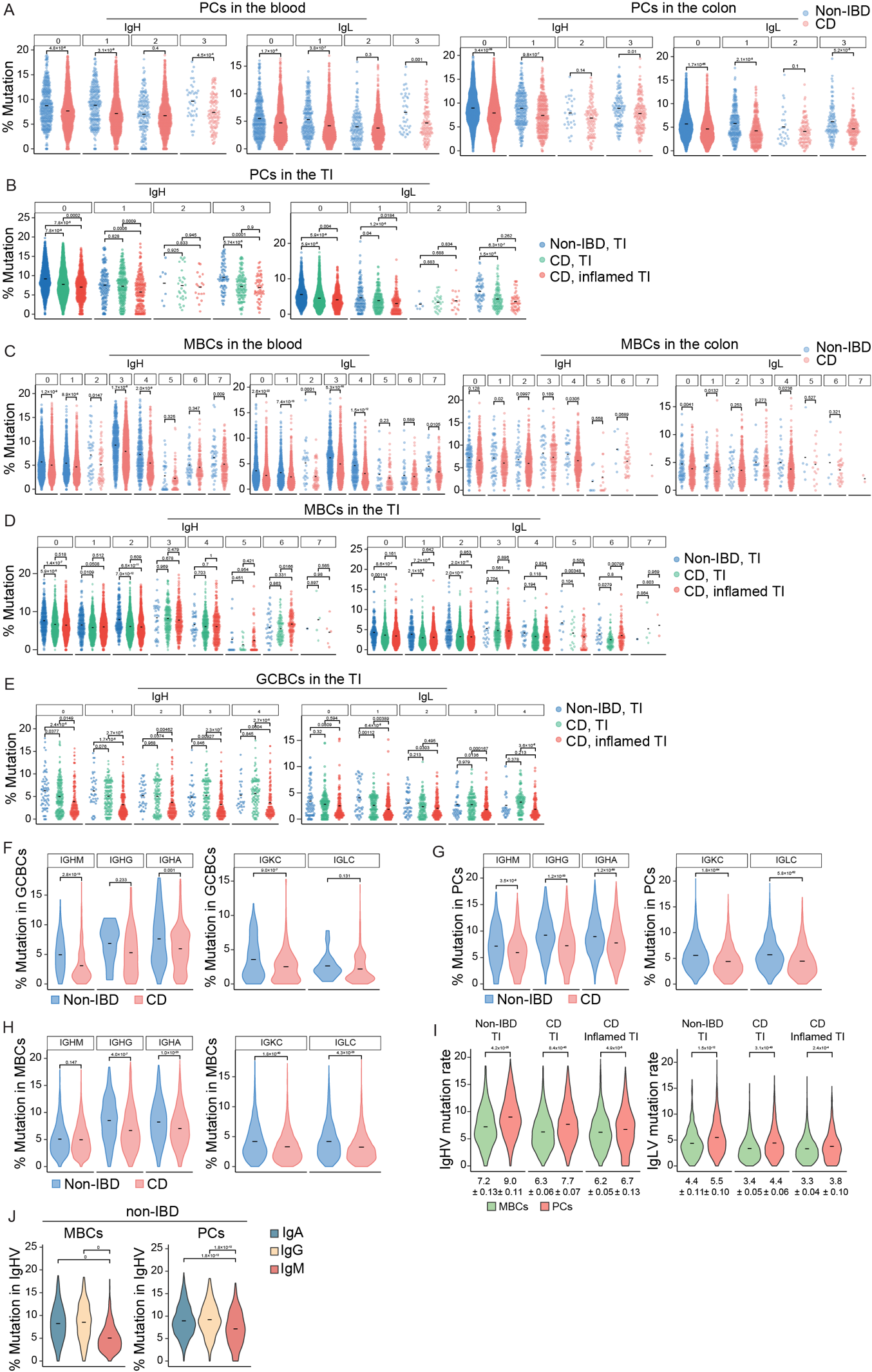
**

**Figure. S8 Reduced Ig somatic hypermutation in CD patients**

**A**,**B**, Quantification of the mutation rate of Ig heavy and light chains in the indicated PC clusters from the blood and colon of non-IBD (blue) and CD (red) patients (A), or in the TI of non-IBD patients (blue) and the visibly healthy (green) or inflamed (red) TI regions of CD patients (B). Dots represent individual cells. Crossbars display the means.

**C**,**D**, Quantification of the mutation rate of Ig heavy and light chains in the indicated MBC clusters from the blood and colon of non-IBD (blue) and CD (red) patients (C), or in the TI of non-IBD patients (blue) and the visibly healthy (green) or inflamed (red) TI regions of CD patients (D). Dots represent individual cells. Crossbars display the means.

**E,** Violin plot showing the mutation rates of Ig heavy and light chains in the indicated GCBC clusters in the TI of non-IBD patients (blue) and the visibly healthy (green) or inflamed (red) TI regions of CD patients. Dots represent individual cells. Crossbars display the means.

**F-H**, Violin plot showing the Ig mutation rates of GCBCs (F), PCs (G) and MBCs (H) with indicated isotypes in non-IBD (blue) and CD (red) patients. Crossbars display the means.

**I**, Violin plot showing the Ig mutation rates of PCs and MBCs in the TI of non-IBD patients and the visibly healthy or inflamed TI regions of CD patients. Numbers display the mean± s.e.m. Crossbars display the means.

**J**, Violin plot showing the distribution of Ig heavy chain variable genes in IgA^+^, IgG^+^ or IgM^+^ MBCs (left) or PCs (right) in non-IBD patients. Crossbars display the means.

*P* values were calculated using two-tailed t-test (A,C,F,G,H,I) or one-way ANOVA with Tukey’s multiple-comparison test (B,D,E,J).

**
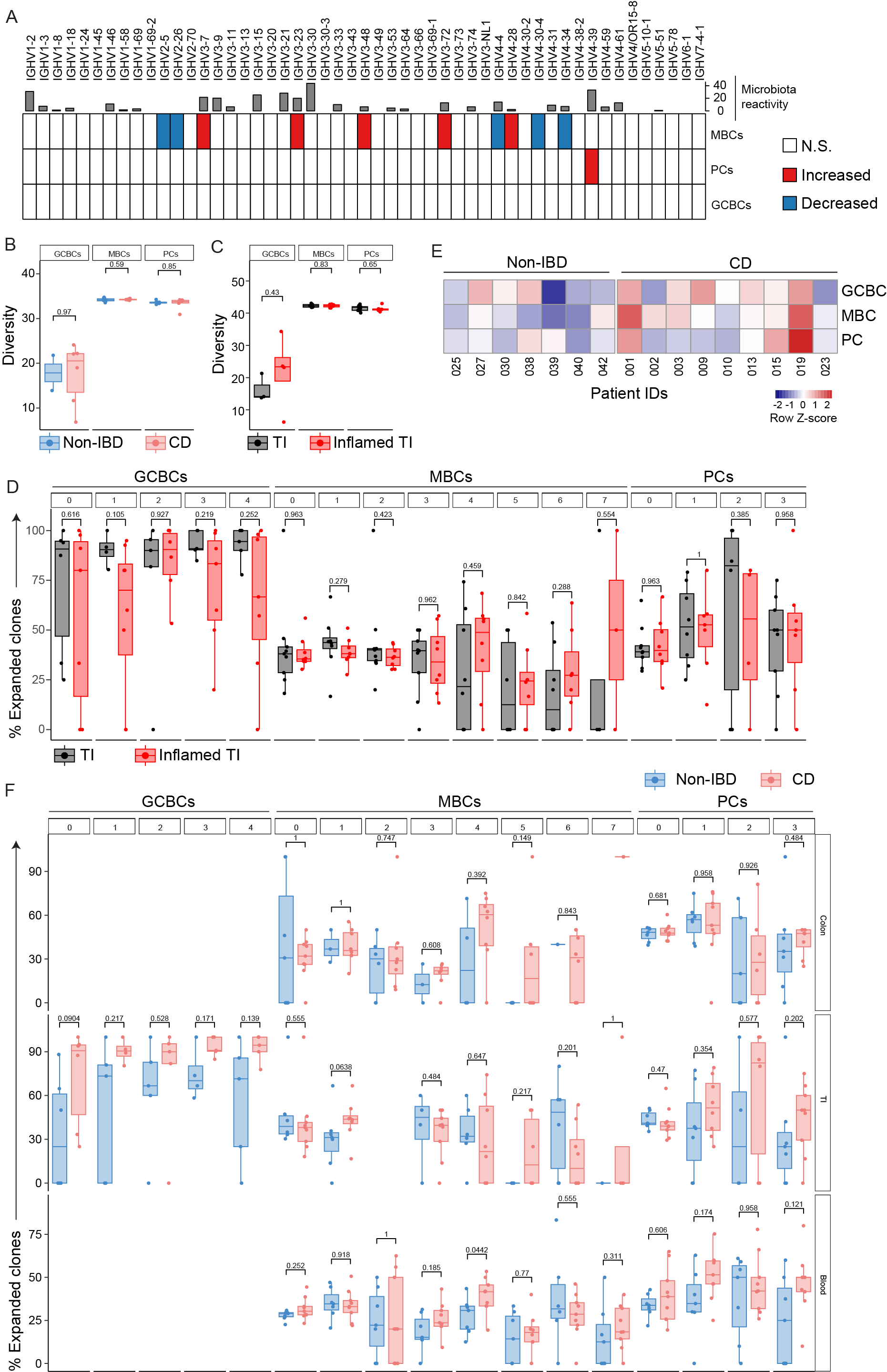
**

**Figure. S9 Mapping Ig diversity, clonal expansion and repertoire similarity**

**A**, Top: bar plot showing the microbiota-binding activities of individual IgHV genes captured in this study. Bottom: heatmap displaying the usage frequency changes of each IgHV gene in the indicated cell types (row) in CD relative to non-IBD patients. Red, significantly increased; blue, significantly decreased; N.S., no significant difference.

**B**, Quantification of the Ig diversity of indicated cell types from non-IBD (blue) and CD (red) patients.

**C**, Quantification of the Ig diversity of indicated cell types from the visibly healthy (black) or inflamed (red) TI regions of CD patients.

**D**, Quantification of the percentages of cells carrying expanded Ig clonotypes in each cluster (column) of the indicated cell types from the visibly healthy (black) or inflamed (red) TI regions of CD patients.

**E**, Heatmap showing the percentages of cells carrying expanded Ig clonotypes in each cell type (row) across individual patients (column). Color: row-wise Z scores.

**F**, Quantification of the percentage of cells carrying expanded Ig clonotypes in each cluster (column) of the indicated cell types in each tissue (row) from non-IBD (blue) and CD (red) patients.

*P* values were calculated using Mann-Whitney test (B,C,D,F).

**
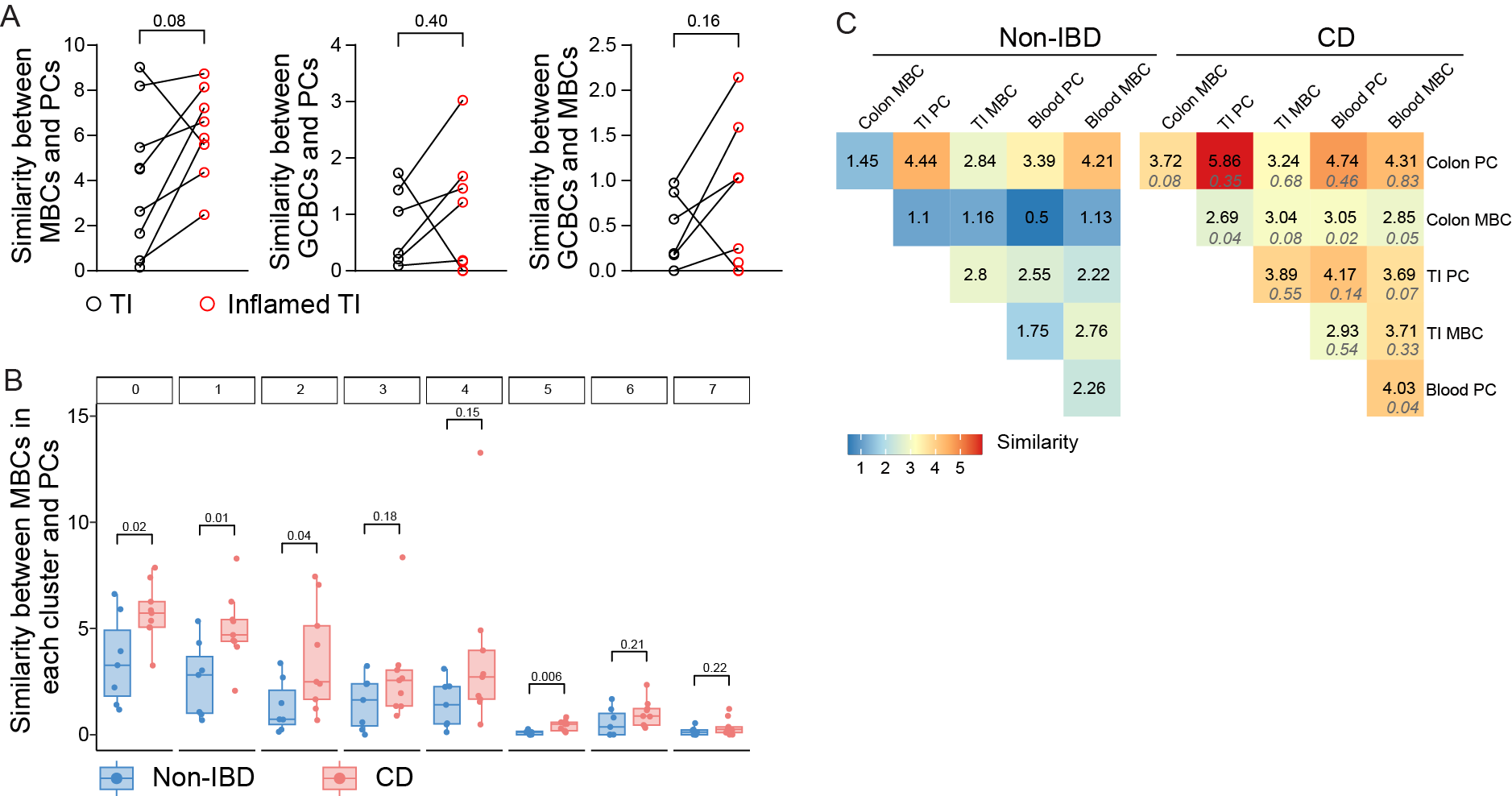
**

**Figure. S10 Ig similarity between different B cell subsets in non-IBD and CD patients**

**A**, Quantification of Ig repertoire similarities between the indicated cell types in the visibly healthy (black) and inflamed (red) TI regions in individual CD patients, connected by lines (n = 8).

**B**, Quantification of Ig similarities between the indicated clusters of MBCs (column) and total PCs from non-IBD (blue) and CD (red) patients.

**C**, Heatmap showing the Ig repertoire similarities between the indicated cell types in each tissue in non-IBD (left) and CD (right) patients.

*P* values were calculated using two-tailed paired t test (A) or Mann-Whitney test (B).

**Supplementary tables**

**Table S1.** Patient characteristics**,** including subject IDs, sample collection time, gender, age, clinical status, clinical diagnosis, time of diagnosis, SES-CD score, current IBD therapy and sampling tissue in five patient cohorts. F, female; M, male; +, the sample was collected; N/A, the sample was not available.

**Table S2.** Gene set enrichment analysis (GSEA) of the GC B cell (GCBC), plasma cell (PC) or memory B cell (MBC) in CD patients compared to non-IBD patients, or in the inflamed terminal ileum (TI) compared to non-inflamed TI.

**Table S3.** List and details of antibodies used for multiparameter flow cytometry

**Table S4.** List of genes used for signature score analysis

**Table S5.** Averaged regulon activities in individual GCBC, PCs or MBC clusters in different tissues from non-IBD and CD patients.
